# Modeling a Controlled-Floating Space Robot for In-Space Services: A Beginner’s Tutorial

**DOI:** 10.3389/frobt.2021.725333

**Published:** 2021-12-24

**Authors:** Asma Seddaoui, Chakravarthini Mini Saaj, Manu Harikrishnan Nair

**Affiliations:** ^1^ Surrey Space Centre, Department of Electronic and Electrical Engineering, University of Surrey, Guildford, United Kingdom; ^2^ Lincoln Centre for Autonomous Systems (L-CAS), University of Lincoln, Lincoln, United Kingdom

**Keywords:** space robot, free-flying, free-floating, controlled-floating, pose control, dynamic modeling

## Abstract

Ground-based applications of robotics and autonomous systems (RASs) are fast advancing, and there is a growing appetite for developing cost-effective RAS solutions for *in situ* servicing, debris removal, manufacturing, and assembly missions. An orbital space robot, that is, a spacecraft mounted with one or more robotic manipulators, is an inevitable system for a range of future in-orbit services. However, various practical challenges make controlling a space robot extremely difficult compared with its terrestrial counterpart. The state of the art of modeling the kinematics and dynamics of a space robot, operating in the free-flying and free-floating modes, has been well studied by researchers. However, these two modes of operation have various shortcomings, which can be overcome by operating the space robot in the controlled-floating mode. This tutorial article aims to address the knowledge gap in modeling complex space robots operating in the controlled-floating mode and under perturbed conditions. The novel research contribution of this article is the refined dynamic model of a chaser space robot, derived with respect to the moving target while accounting for the internal perturbations due to constantly changing the center of mass, the inertial matrix, Coriolis, and centrifugal terms of the coupled system; it also accounts for the external environmental disturbances. The nonlinear model presented accurately represents the multibody coupled dynamics of a space robot, which is pivotal for precise pose control. Simulation results presented demonstrate the accuracy of the model for closed-loop control. In addition to the theoretical contributions in mathematical modeling, this article also offers a commercially viable solution for a wide range of in-orbit missions.

## 1 Introduction

Innovative space system technologies have revolutionized the lives on Earth, which explains why the global space race continues. The space economy worldwide is booming, and there is now a paradigm shift to the in-orbit services and manufacturing (IOSM) market. There is a significant commercial boost in the in-orbit services market, which is predicted to be $1 billion by 2030 (Satellite Catapult Applications, Fair-Space and Astroscale, 2021). The candidate in-orbit missions include the following: servicing and repairing high-value space assets including operational spacecraft, life extension, refueling, orbit correction, in-space assembly of space telescopes for Earth observation and astronomical observations, space-based power generation, and active debris removal, to name a few (Oda et al., 1996; Whittaker et al., 2000; Yoshida, 2001; Friend, 2008; Shengwei, 2013; Flores-Abad et al., 2014; Jaekel et al., 2015; Lee et al., 2016; Reed et al., 2016; Medina et al., 2017; Taylor et al., 2018; Li et al., 2019; Wilde et al., 2019; Jackson et al., 2020; Nair et al., 2020; Romano, 2021; Xu, 2021). Building satellite servicing and debris removal capabilities will open up bigger and longer-term markets linked to assembly and manufacturing in space (Satellite Catapult Applications, Fair-Space and Astroscale, 2021). As the in-orbit economy evolves, robotics and autonomous systems will play a pivotal role in many future IOSM missions. However, controlling a space robot in an extreme environment is significantly more complex than its terrestrial counterpart; this is the foremost hurdle to the success of IOSM missions.

In the context of servicing a cooperative and noncooperative target spacecraft using a servicer space robot, safety is paramount during the close-range approach, target capture, and postcapture operations. Approaching a target spacecraft using a servicer space robot can be achieved by two different modes of operation: free-flying and free-floating (Dubowsky and Papadopoulos, 1993; Moosavian and Papadopoulos, 2007). The free-flying approach uses reaction jets to facilitate a stabilized and controlled base for the robot manipulator in motion. The stable platform is favorable for the manipulator’s motion, however, on the expense of excessive fuel consumption and limited workspace. On the other hand, the free-floating approach utilizes an uncontrolled base to limit fuel consumption on the expense of dynamic singularities and an undefined workspace for the robot. There is a well-established literature on these two modes of operation, and they have benefits depending on the nature of the mission (Longman et al., 1987; Papadopoulos and Dubowsky, 1991, 1993; Xu and Kanade, 1992; Xu, 1993; Yoshida and Abiko, 2002; Menon et al., 2007; Artigas et al., 2015; Guang et al., 2018; Wilde et al., 2018). For instance, when communication between the space robot and the ground station is paramount, the free-flying mode is preferred as its controlled base can keep the antennas pointing toward the Earth. On the other hand, when reducing fuel consumption is prioritized, the free-floating mode is more suitable because of its uncontrolled base. In addition, recent pertinent works include numerical simulations, hardware-in-the-loop experiments, and guidance algorithms to capture and detumble a space object (Virgili-Llop et al., 2017). Despite these current advancements, the free-flying and free-floating operation modes exhibit unavoidable disadvantages for practical in-orbit missions.

An inherent problem with free-flying and free-floating operation modes is the undesired dynamic coupling effect due to the manipulator’s motion. This dynamic coupling effect leads to changes in the pose of the spacecraft base that are not always corrected, depending on the selected approach mode. In the free-flying mode, reaction jets will control the spacecraft base to maintain a fixed pose or attain one alternate pose while the arm is in motion. On the other hand, in the free-floating mode, the spacecraft base is uncontrolled, and it is free to change its pose in reaction to the motion of the arm. The free-flying mode has a limited workspace, whereas the free-floating mode has an undefined workspace because of its uncontrolled base. As a result, it is highly challenging to precisely navigate and control the space robot while avoiding obstacles and singularities during the approach phase.

In the close vicinity of the target, well-defined trajectories for the position and attitude of the spacecraft base are needed along with the trajectories for the arm’s joints to perform collision-free navigation. Instead of maintaining a fixed pose for the base spacecraft or letting the base spacecraft float in an uncontrolled manner, a controlled motion of the spacecraft base (i.e., achieving desired translation and rotation with time) is highly desirable. A coordinated movement of this nature is distinct and challenging compared with both free-flying and free-floating modes. It corresponds to the “controlled-floating” mode, previously introduced by Seddaoui and Saaj (2019), which offers redundancy to operate the space robot in an unlimited but well-defined workspace. The space robot is referred to as the controlled-floating space robot (CFSR) when operated in this mode. Contrarily to the free-flying and free-floating space robots, the CFSR uses its base’s controlled translation and rotation to help the arm reach the target’s grasping point.

Literature review shows that space robots suffer from singularities, depending on the mode of operation. It is also known that dynamic singularities affect free-floating space robots, whereas kinematic singularities affect free-flying space robots. As the CFSR can control its base’s motion, only kinematic singularities occur during the arm’s movement. It is possible to avoid kinematic singularities using the extra degrees of freedom offered by the spacecraft base. In short, the controlled-floating mode offers the benefits of both free-flying and free-floating modes of operation, and an accurate model of a CFSR will be highly beneficial to the end-users.

Although modeling the kinematics and dynamics of a free-flying and free-floating space robots is well addressed in the literature, the same for CFSR is less extensively studied in literature. Recently, Virgili-Llop et al. (2019) and Virgili-Llop and Romano (2019) have obtained simulation and experimental results for a CFSR approach via convex programming for maneuvering and capturing a tumbling object. In order to help the beginners in the discipline and foster an increased use of the CFSR approach, this article presents a step-by-step tutorial on deriving the equation of motion of a CFSR during its final approach phase. The full-scale nonlinear model presented can be used for controlling the pose of a CFSR during any of the aforementioned in-orbit missions. This article’s benefits are twofold: (1) helps beginners gain a sound theoretical foundation in mathematical modeling of orbital robots and (2) creates a better awareness of the benefits of CFSR for practical space missions.

Compared with the mathematical models of free-flying and free-floating space robots reported in the literature, the model of CFSR shown in this article includes a few new terms that represent different types of internal and external perturbations. More specifically, in this article, the motion of a CFSR is derived with respect to a reference frame attached to the moving target; thus, it complies with the close-proximity relative motion in-orbit. The center of mass (CoM) of the space robot changes because of both the arm and the base spacecraft’s motion. In addition, the overall inertia matrix at the CoM of the system is also not constant, but it changes during the motion of the CFSR. Corresponding changes in CoM, inertia matrix, Coriolis, and centrifugal terms are modeled in addition to accounting for the dynamic coupling effect. An accurate dynamic model together with a robust controller will enable the space robot, operating in the controlled-floating mode, to perform a safe and precise maneuver to approach the grasping point on the target. More details on the control architecture for a CFSR were published by Seddaoui and Saaj (2019).

In summary, the model of the CFSR presented in this article advances the state of the art of dynamic modeling a space robot. In addition to this article’s theoretical contributions, the highly accurate model presented is well suited for practical in-orbit missions. The rest of this article is organized as follows: Section 2 gives a recap of the CFSR and its mode of operation. The detailed tutorial that explains the mathematical modeling is covered in Section 3. The accuracy of the model presented is verified using simulations, and selected results are shown in Section 4. Finally, Section 5 summarizes the key inferences of the modeling methodology and added value of model presented for controlling the CFSR in orbit.

## 2 Background on CFSR

The concept of a CFSR originated with the idea of fusing the free-flying and free-floating operation modes of a space robot to utilize their individual pros. Both these modes of operation, individually, are undesirable for in-space services, which require precise operations; a limited or undefined workspace is unsatisfactory. Therefore, the identifiable challenges include collision-free navigation of the space robot with efficient fuel consumption.

A CFSR addresses these practical challenges by utilizing a closed-loop control for the motion of the space robot. Therefore, in addition to the arm’s joints, well-defined trajectories are needed to maintain the position and attitude of the spacecraft base. These desired trajectories are generated based on the linear and angular motion of the target spacecraft. In addition, the CFSR also takes into account the reaction forces and moments developed due to the actuation of the robotic arm. The dynamic coupling effect has to be controlled to withstand the undamped vibrations in the extremities of the space environment. The servicer spacecraft should match angular rates and keep zero relative attitude between the target to avoid misalignments.

**FIGURE 1:**
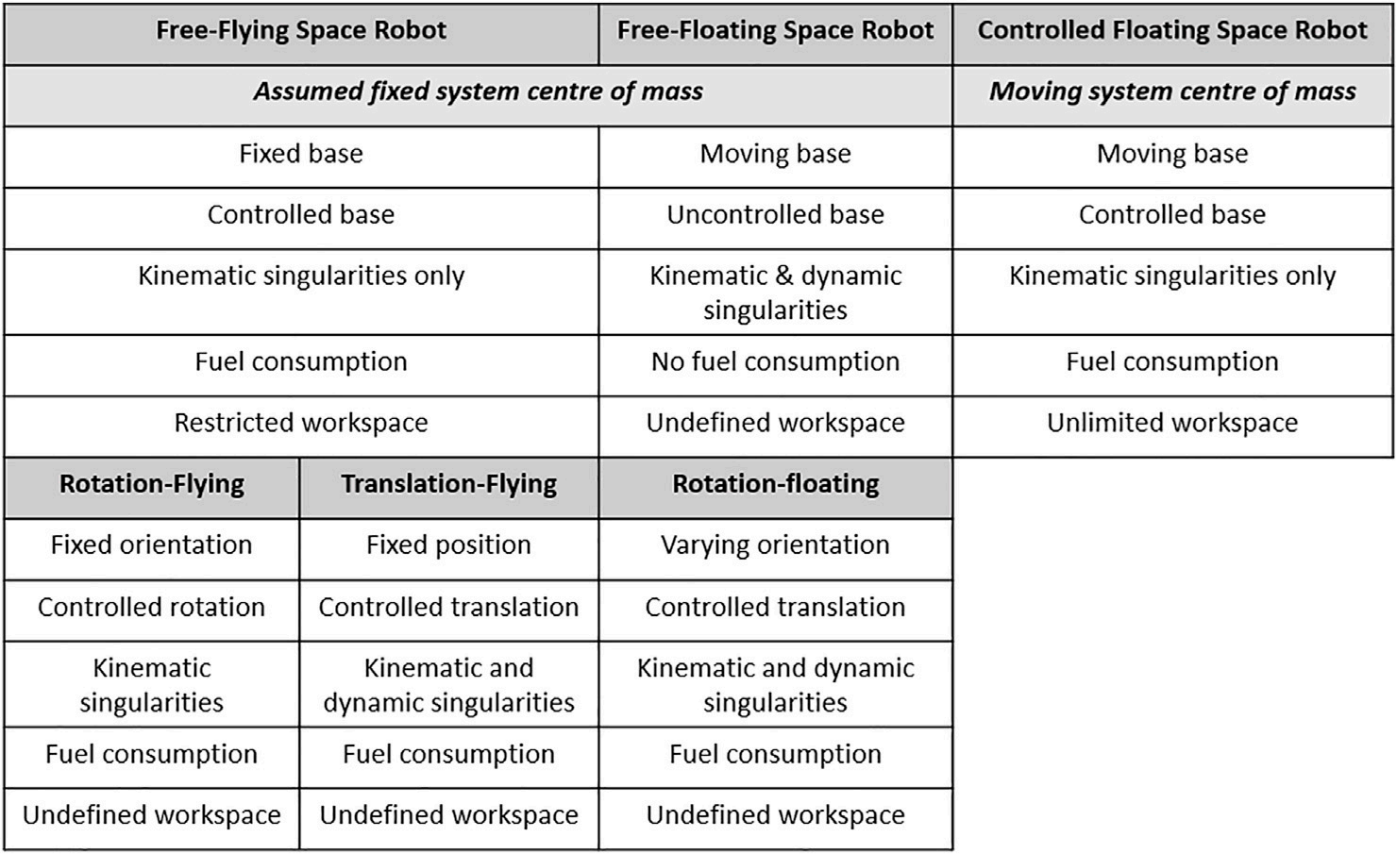
Comparison between existing modes of operation for space robot and the CFSR.

Seddaoui and Saaj (2019) introduced the CFSR system first, but there is limited literature on its dynamic modeling. This tutorial aims at explaining the complex dynamics of CFSR, through a systematic mathematical formulation, at a high level of granularity. The details of path planning and robust control of CFSR are outside this article’s scope; details are available in Seddaoui (2020). A comparison between the existing modes of operation and the CFSR is required before any derivation. Figure 1 shows a comparison between the two main existing modes of operation, that is, free-flying and free-floating, as well as their subcategories introduced by Wilde et al. (2018) against the CFSR.

### 2.1 In-Orbit Relative Motion for the CFSR

The literature review reveals that modeling a space robot, which has a controlled spacecraft base, is always performed with respect to an inertial reference frame (Moosavian and Papadopoulos, 2007; Flores-Abad et al., 2014). This is to enable the utilization of Newton’s law of motion to derive the equation of motion of the space robot. Nevertheless, using a space robot for an in-orbit close-proximity approach is described as a three-body problem where it is preferable to reference the motion of the chasing body with respect to the moving and rotating frame of the target body, also known as local vertical local horizontal (LVLH) frame. Then it is necessary to transform the motion in LVLH back to the inertial frame to find the true actuating forces.

**FIGURE 2:**
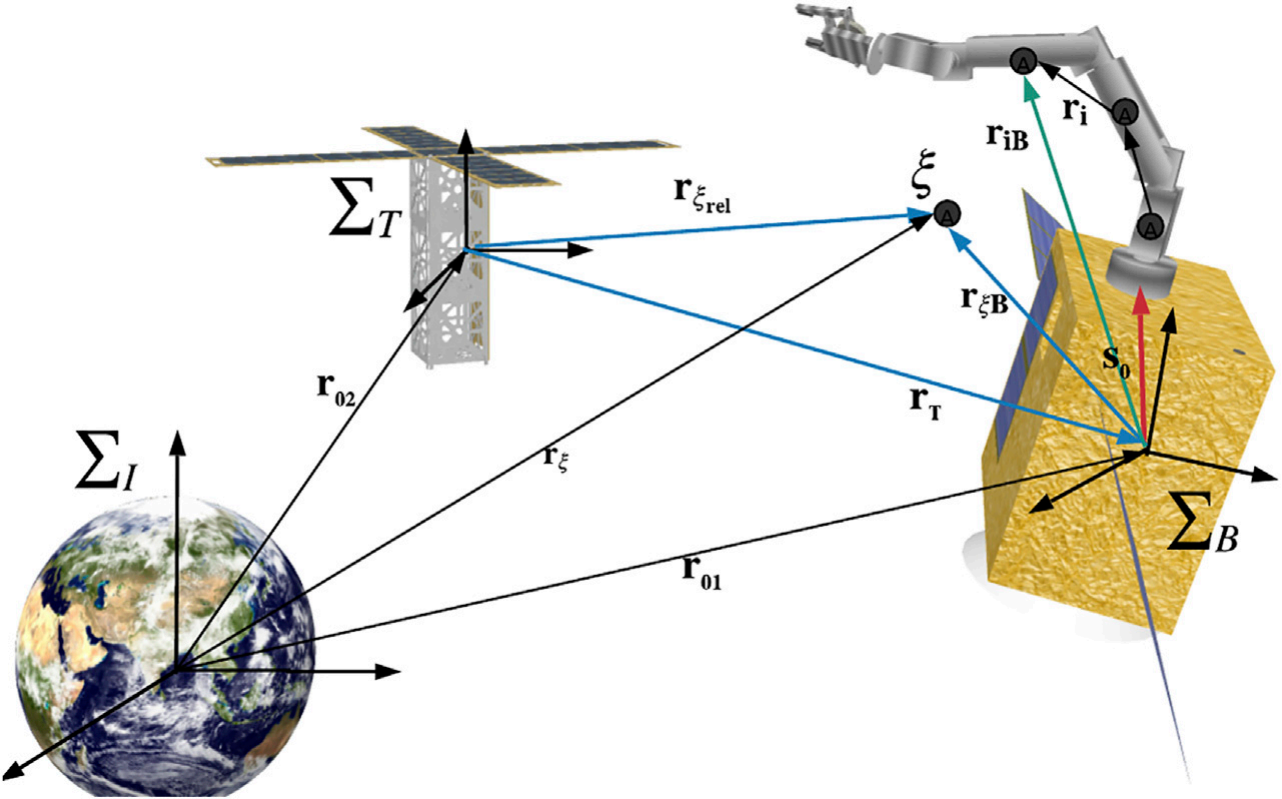
Artistic illustration of the reference frames and vectors used in the mathematical model for the CFSR (Seddaoui, 2020).

In this article, under the assumption that the space robot is operated at a very close proximity to the target (<10 m) and considering a short period (up to 150 s), three frames of reference are selected to derive the equation of motion of the CFSR as shown in Figure 2: the inertial frame *∑*_*I*_, the target LVLH frame *∑*_*T*_, and the base of the CFSR body frame *∑*_*B*_. The idea is to consider the motion of the CFSR in *∑*_*T*_ as the controllable quantity in the closed-loop control and compute the fictitious forces, originating from the rotating frame *∑*_*T*_, as external forces affecting the system. This is to ensure that the true actuating forces and torques are identified in *∑*_*I*_.

The motion of the CFSR is described about its overall CoM, referred to here as *ξ*. Hence, the relative position is defined as the vector between the origin of *∑*_*T*_ and the CoM *ξ*, referred to as $r_{\xi_{rel}}$ . The basic equation of a relative position vector $r_{\xi_{rel}}$ in *∑*_*I*_ is (Curtis, 2013):

$$r_{\xi_{rel}}=r_{\xi }-r_{02},$$ (1)

where vector $r_{\xi }\in R^{3}$ represents the inertial position vector of the CoM of the space robot *ξ*, $r_{02}\in R^{3}$ is the inertial position vector of the target, and $r_{\xi_{rel}}\in R^{3}$ is the relative position vector between *ξ* and the origin of *∑*_*T*_, as shown in Figure 2. The corresponding relative velocity and acceleration vectors in *∑*_*I*_ are defined as follows (Curtis, 2013):

$$\begin{array}{ll} v_{\xi }-v_{02} & ={\dot{r}}_{\xi_{rel}}+\Omega \times r_{\xi_{rel}} \end{array}$$ (2a)

$$\begin{array}{ll} a_{\xi }-a_{02} & ={\ddot{r}}_{\xi_{rel}}+\dot{\Omega }\times r_{\xi_{rel}}+\Omega \times \left(\Omega \times r_{\xi_{rel}}\right)+2\Omega \times {\dot{r}}_{\xi_{rel}} \end{array}$$ (2b)

where $\Omega \in R^{3}$ is the angular velocity of the rotating frame *∑*_*T*_ with respect to *∑*_*I*_; $v_{\xi }\in R^{3}$ and $v_{02}\in R^{3}$ are the velocity vectors of the CoM *ξ* and the target, respectively; $a_{\xi }\in R^{3}$ and $a_{02}\in R^{3}$ are the acceleration vectors of the CoM *ξ* and the target, respectively. In the above equation and thereafter in this article, as it is often customary in the robotics literature, we use the word “vector” to indicate the column matrix of three scalar components of a physical vector in a specific Cartesian triad of axes. Furthermore, we use the symbol of vector cross product in order to indicate the three-by-three skew-symmetric matrix expressing in matricial form the cross product and built with the components of the algebraic vector preceding that symbol.

Eventually, the state vector that will be used in the closed-loop control of the CFSR includes the relative motion between the space robot and the target described by $r_{\xi_{rel}}$ , ${\dot{r}}_{\xi_{rel}}$ and ${\ddot{r}}_{\xi_{rel}}$ . The fictitious velocity and acceleration involved in Eqs 2a, 2b are to be added as extra motion generated from the motion of *∑*_*T*_ in order to find the true actuating forces and torques in *∑*_*I*_. The motion of the space robot’s base has both linear and angular components, as well as the generated interactions between the translational and rotational motion. This is computed through deriving the space robot’s motion about the overall CoM *ξ*.

## 3 Spacecraft Motion

The motion of the space robot’s base has both linear and angular components, as well as the generated interactions between the translational and rotational motion. This is computed through deriving the space robot’s motion about the overall CoM *ξ*.

### 3.1 The Linear Motion

During close-proximity maneuvers, the relative motion is of interest. Hence, the linear motion of the spacecraft base consists of its own relative motion, as well as that of the arm. It is translated by the relative linear momentum of the system as follows:

$$\left.\begin{array}{ccc} P & = & \sum_{i=0}^{n}m_{i}\dot{r_{i}}\\ P & = & M_{t}\left(v_{\xi }-v_{02}\right) \end{array}\right\},$$ (3)

where $P\in R^{3}$ is the linear momentum of the system; *m*_*i*_ and ***r***_***i***_ are, respectively, the mass and the position vector of the *i*th link of the space robot including the spacecraft base; and *M*_*t*_ is the total mass of the system. Differentiating Eq. 3and substituting Eq. 2b into Eq. 3 gives the equation describing the relative linear motion of the spacecraft base about the CoM (*ξ*) with respect to *∑*_*I*_:

$$\left.\begin{array}{cc} F_{sc}^{I} & =M_{t}\left(a_{\xi }-a_{02}\right)\\ & =M_{t}{\ddot{r}}_{\xi_{rel}}+M_{t}\dot{\Omega }\times r_{\xi_{rel}}+M_{t}\Omega \times \left(\Omega \times r_{\xi_{rel}}\right)+2M_{t}\Omega \times {\dot{r}}_{\xi_{rel}} \end{array}\right\},$$ (4)

where $F_{sc}^{I}\in R^{3}$ is the force acting on the spacecraft base about *ξ* in *∑*_*I*_. The CoM of the full system *ξ* is different from the CoM of the base spacecraft. Also, the actuation forces and torques are applied on the spacecraft base rather than *ξ*. Therefore, in order to derive the equation of the relative linear motion of the spacecraft base, one needs to introduce the motion of *∑*_*B*_ in *∑*_*T*_. Hence, using the equation for the relative velocity and acceleration, as seen in Eqs 2a, 2b, gives the equation for the relative velocity and acceleration ${\dot{r}}_{\xi_{rel}}\in R^{3}$ and ${\ddot{r}}_{\xi_{rel}}\in R^{3}$ when considering the motion of *∑*_*B*_. In other words, the following represents the expression of the first term of Eqs 2a, 2b when introducing the motion of *∑*_*B*_:

$$\begin{array}{ll} r_{\xi_{rel}} & =r_{T}+r_{\xi B} \end{array}$$ (5a)

$$\begin{array}{ll} {\dot{r}}_{\xi_{rel}} & ={\dot{r}}_{T}+{\dot{r}}_{\xi B}+\omega_{sc}\times r_{\xi B} \end{array}$$ (5b)

$$\begin{array}{ll} {\ddot{r}}_{\xi_{rel}} & ={\ddot{r}}_{T}+{\ddot{r}}_{\xi B}+{\dot{\omega }}_{sc}\times r_{\xi B}+\omega_{sc}\times \left(\omega_{sc}\times r_{\xi B}\right)+2\omega_{sc}\times {\dot{r}}_{\xi B} \end{array}$$ (5c)

where $r_{T}\in R^{3}$ is the relative position vector of the spacecraft base defined as $r_{T}=r_{01}-r_{02}$ , vector $r_{\xi B}\in R^{3}$ represents the relative position vector between *ξ* and *∑*_*B*_, and vector $\omega_{sc}\in R^{3}$ is the angular velocity of the spacecraft base with respect to *∑*_*T*_. Multiplying Eq. 5c by the total mass of the system gives the following expression for the linear motion of the spacecraft base in *∑*_*T*_, which is function of the spacecraft base’s position vector $r_{T}$ instead of the overall CoM’s position vector $r_{\xi_{rel}}$ , as follows:

$$F_{sc}=M_{t}{\ddot{r}}_{T}+M_{t}{\ddot{r}}_{\xi B}-M_{t}r_{\xi B}\times {\dot{\omega }}_{sc}-M_{t}\left(\omega_{sc}\times r_{\xi B}\right)\times \omega_{sc}+2M_{t}\omega_{sc}\times {\dot{r}}_{\xi B},$$ (6)

where $F_{sc}\in R^{3}$ represents the force acting on the spacecraft base about *ξ* in *∑*_*T*_.

Substituting Eqs 5a, 5b into the last three remaining terms of Eq. 4 gives the following expression for the fictitious forces resulting from the rotation of frame *∑*_*T*_:

$$\begin{array}{cc} F_{sc}^{fict} & =M_{t}\dot{\Omega }\times \left(r_{T}+r_{\xi B}\right)+M_{t}\Omega \times \left(\Omega \times \left({\dot{r}}_{T}+{\dot{r}}_{\xi B}\right)\right)\\ & +2M_{t}\Omega \times \left({\dot{r}}_{T}+{\dot{r}}_{\xi B}+{\dot{\omega }}_{sc}\times r_{\xi B}\right), \end{array}$$ (7)

where $F_{sc}^{fict}\in R^{3}$ represents the virtual forces generated from the rotation of the frame *∑*_*T*_.

Substituting Eqs 6, 7 into Eq. 4 gives the relative linear motion of the spacecraft base in *∑*_*I*_ when considering the motion of frame *∑*_*T*_. This is expressed as follows:

$${F_{sc}}^{I}=F_{sc}+{F_{sc}}^{fict}.$$ (8)

The first term of Eq. 8 represents the controlled linear motion of the space robot with respect to *∑*_*T*_. The second term is related to the rotation of *∑*_*T*_, which must be included in the equation of motion to account for the virtual forces that exist only in the rotating frame of reference. Expressing the linear motion of the space robot, represented by Eq. 6, in matrix form gives:

$$\begin{array}{cc} F_{sc} & =\left[\begin{array}{cc} M_{t}E & -M_{t}{\left[r_{\xi B}\right]}_{\times } \end{array}\right]\left[\begin{array}{c} {\ddot{r}}_{T}\\ {\dot{\omega }}_{sc} \end{array}\right]+\left[\begin{array}{cc} 0 & -M_{t}{\left[\omega_{sc}\times r_{\xi B}\right]}_{\times } \end{array}\right]\left[\begin{array}{c} {\dot{r}}_{T}\\ \omega_{sc} \end{array}\right]\;+\\ & +\;M_{t}{\ddot{r}}_{\xi B}+2M_{t}\omega_{sc}\times {\dot{r}}_{\xi B}, \end{array}$$ (9)

where $E\in R^{3\times 3}$ is the identity matrix. Eq. 9 describing the relative motion of the spacecraft base is one of the components constituting the equation of the relative motion of the CFSR.

### 3.2 The Angular Motion

The angular momentum of the multibody space robot about *ξ*, in terms of the inertia tensor and the angular velocity, is defined, with respect to a frame attached to the CoM of the spacecraft base and that does not rotate with the *∑*_*B*_, as follows (see Supplementary Material):

$$L_{\xi }=I_{\xi }\omega_{\xi }+\overset{n}{\underset{i=0}{\sum }}I_{i}\omega_{i}+\overset{n}{\underset{i=0}{\sum }}\left(I_{i}-m_{i}{\left[r_{iB}\right]}_{\times }{\left[r_{iB}\right]}_{\times }\right)\omega_{\xi B}$$ (10)

where $I_{\xi }\in R^{3\times 3}$ and $I_{i}\in R^{3\times 3}$ are, respectively, the inertia tensors of the full system expressed at the CoM *ξ* and the *i*th link of the arm; ***ω***_***ξ***_ is the angular velocity about *ξ* with respect to a frame attached at the origin of *∑*_*B*_; and ***ω***_***ξB***_ is the angular velocity about *ξ* with respect to *∑*_*B*_.

Let $I_{iB}=\sum_{i=1}^{n}\left(I_{i}-m_{i}{\left[r_{iB}\right]}_{\times }{\left[r_{iB}\right]}_{\times }\right)$ , then Eq. 10 becomes:

$$L_{\xi }=I_{\xi }\omega_{\xi }+\overset{n}{\underset{i=1}{\sum }}I_{i}\omega_{i}+I_{iB}\omega_{\xi B}$$ (11)

Given ***ω***_***ξ***_ = ***ω***_***sc***_ + ***ω***_***ξB***_, Eq. 10 becomes:

$$L_{\xi }=I_{\xi }\omega_{sc}+I_{\xi }\omega_{\xi B}+\overset{n}{\underset{i=1}{\sum }}I_{i}\omega_{i}+I_{iB}\omega_{\xi B}.$$ (12)

The second and last terms of Eq. 12 are related to the rotation about *ξ*, generated by the motion of the arm. Differentiating Eq. 12 gives:

$$\begin{array}{c} {\dot{L}}_{\xi }=\frac{d}{dt}\left(I_{\xi }\omega_{sc}\right)+\overset{n}{\underset{i=1}{\sum }}\frac{d}{dt}\left(I_{i}\omega_{i}\right)+\frac{d}{dt}\left(I_{\xi }\omega_{\xi B}\right)+\frac{d}{dt}\left(I_{iB}\omega_{\xi B}\right). \end{array}$$ (13)

As the CFSR is one redundant system, finding the total inertia tensor **I**_***ξ***_ involves two steps: use the parallel axis theorem to express the total inertia matrix of both the spacecraft base and the arm at the origin of *∑*_*B*_ and then express the result at *ξ*. The total inertia matrix at the origin of *∑*_*B*_ is as follows:

$$I_{\xi B}=I_{sc}+\overset{n}{\underset{i=1}{\sum }}\left(I_{i}-m_{i}{\left[r_{iB}\right]}_{\times }{\left[r_{iB}\right]}_{\times }\right).$$ (14)

Considering the space robot with its overall CoM *ξ*, which is different from the CoM of the spacecraft base alone, the total inertia tensor of the system at *ξ*, using the parallel axis theorem, is:

$$I_{\xi }=I_{\xi B}+M_{t}{\left[r_{\xi B}\right]}_{\times }{\left[r_{\xi B}\right]}_{\times }.$$ (15)

Substituting Eq. 14 into Eq. 15 gives:

$$\left.\begin{array}{cc} I_{\xi } & =I_{sc}+M_{t}{\left[r_{\xi B}\right]}_{\times }{\left[r_{\xi B}\right]}_{\times }+\sum_{i=1}^{n}\left(I_{i}-m_{i}{\left[r_{iB}\right]}_{\times }{\left[r_{iB}\right]}_{\times }\right)\\ & =I_{sc}+M_{t}{\left[r_{\xi B}\right]}_{\times }{\left[r_{\xi B}\right]}_{\times }+I_{iB} \end{array}\right\}.$$ (16)

It is clear from Eq. 16 that the inertia tensor of the space robot is not constant as it depends on the position of the moving CoM *ξ* and the moving arm’s links. Hence, the derivatives of the first, second, and third terms of Eq. 13 are given as follows:

$$\begin{array}{ll} \frac{d}{dt}\left(I_{\xi }\omega_{sc}\right) & =I_{\xi }{\dot{\omega }}_{sc}+{\dot{I}}_{\xi }\omega_{sc}, \end{array}$$ (17a)

$$\begin{array}{ll} \overset{n}{\underset{i=1}{\sum }}\frac{d}{dt}\left(I_{i}\omega_{i}\right) & =\overset{n}{\underset{i=1}{\sum }}I_{i}{\dot{\omega }}_{i} \end{array}$$ (17b)

$$\begin{array}{ll} \frac{d}{dt}\left(I_{\xi }\omega_{\xi B}\right) & =I_{\xi }{\dot{\omega }}_{\xi B}+{\dot{I}}_{\xi }\omega_{\xi B} \end{array}$$ (17c)

$$\begin{array}{ll} \frac{d}{dt}\left(I_{iB}\omega_{\xi B}\right) & =I_{iB}\dot{\omega_{\xi B}}+{\dot{I}}_{iB}\omega_{\xi B} \end{array}$$ (17d)

The derivatives of the inertia tensors **I**_***ξ***_ and **I**_**iB**_ (see Supplementary Material) still appear in the equation because they are function of position vectors that vary during the motion of the space robot, as seen in Eq. 16.

Substituting 17c and 17d into Eq. 13 gives the equation for the rotational motion of the space robot about *ξ* as follows:

$$\begin{array}{cc} \tau_{sc}^{\prime } & ={\dot{L}}_{\xi }=I_{\xi }{\dot{\omega }}_{sc}+{\dot{I}}_{\xi }\omega_{sc}+\sum_{i=0}^{n}I_{i}{\dot{\omega }}_{i}+I_{\xi }{\dot{\omega }}_{\xi B}+{\dot{I}}_{\xi }\omega_{\xi B}\;+\\ & +I_{iB}{\dot{\omega }}_{\xi B}+{\dot{I}}_{iB}\omega_{\xi B} \end{array}$$ (18)

Eq. 18 describes the rotational dynamics of the space robot about the overall CoM *ξ* with respect to *ξ*. Expressing this vector of torques relative to the CoM of the spacecraft base (origin of *∑*_*B*_), with respect to *∑*_*B*_, requires the following transformation (Curtis, 2013):

$$\tau_{sc}^{I}=\tau_{sc}^{\prime }+r_{\xi B}\times F_{sc}^{I}.$$ (19)

Substituting Eqs 8–18 into Eq. 19 and simplifying give:

$$\begin{array}{cc} \tau_{sc}^{I} & =M_{t}{\left[r_{\xi B}\right]}_{\times }{\ddot{r}}_{T}+\left(I_{sc}+I_{iB}\right){\dot{\omega }}_{sc}-M_{t}r_{\xi B}\times \left(\left(\omega_{sc}\times r_{\xi B}\right)\times \omega_{sc}\right)\;+\\ & +\;{\dot{I}}_{\xi }\omega_{sc}+\sum_{i=0}^{n}I_{i}{\dot{\omega }}_{i}+{\dot{I}}_{\xi }\omega_{\xi B}+I_{\xi }{\dot{\omega }}_{\xi B}+{\dot{I}}_{iB}\omega_{\xi B}+I_{iB}{\dot{\omega }}_{\xi B}\\ & +\;+M_{t}r_{\xi B}\times {\ddot{r}}_{\xi B}+2M_{t}r_{\xi B}\times \left(\omega_{sc}\times {\dot{r}}_{\xi B}\right)+r_{\xi B}\times F_{sc}^{fict}. \end{array}$$ (20)

From Eq. 20, the angular motion of the space robot in *∑*_*T*_ can be represented in matrix form as follows:

$$\begin{array}{cc} \tau_{sc} & =\left[\begin{array}{cc} M_{t}{\left[r_{\xi B}\right]}_{\times } & I_{sc}+I_{iB} \end{array}\right]\left[\begin{array}{c} {\ddot{r}}_{T}\\ {\dot{\omega }}_{sc} \end{array}\right]\;\\ & +\;\left[\begin{array}{cc} 0 & {\dot{I}}_{\xi }\omega_{sc}-M_{t}{\left[r_{\xi B}\right]}_{\times }{\left[\omega_{sc}\times r_{\xi B}\right]}_{\times } \end{array}\right]\left[\begin{array}{c} {\dot{r}}_{T}\\ \omega_{sc} \end{array}\right]\;\\ & +\;\sum_{i=0}^{n}I_{i}{\dot{\omega }}_{i}+{\dot{I}}_{\xi }\omega_{\xi B}+I_{\xi }{\dot{\omega }}_{\xi B}+{\dot{I}}_{iB}\omega_{\xi B}+I_{iB}{\dot{\omega }}_{\xi B}\\ & +\;M_{t}r_{\xi B}\times {\ddot{r}}_{\xi B}+2M_{t}r_{\xi B}\times \left(\omega_{sc}\times {\dot{r}}_{\xi B}\right). \end{array}$$ (21)

### 3.3 The Overall Motion of the Spacecraft Base

From Eqs 9–21, the overall equation describing the linear and angular motion of the spacecraft base in *∑*_*T*_ is:

$$\begin{array}{cc} f_{sc} & =\left[\begin{array}{c} F_{sc}\\ \tau_{sc} \end{array}\right]=\left[\begin{array}{cc} M_{t}E & -M_{t}{\left[r_{\xi B}\right]}_{\times }\\ M_{t}{\left[r_{\xi B}\right]}_{\times } & I_{sc}+I_{iB} \end{array}\right]\left[\begin{array}{c} {\ddot{r}}_{T}\\ {\dot{\omega }}_{sc} \end{array}\right]\;+\\ & +\;\left[\begin{array}{cc} 0 & -M_{t}{\left[\omega_{sc}\times r_{\xi B}\right]}_{\times }\\ 0 & {\dot{I}}_{\xi }\omega_{sc}-M_{t}{\left[r_{\xi B}\right]}_{\times }{\left[\omega_{sc}\times r_{\xi B}\right]}_{\times } \end{array}\right]\left[\begin{array}{c} {\dot{r}}_{T}\\ \omega_{sc} \end{array}\right]\;+\\ & \;+\;\left[\begin{array}{c} M_{t}{\ddot{r}}_{\xi B}\\ M_{t}r_{\xi B}\times {\ddot{r}}_{\xi B}+\sum_{i=0}^{n}I_{i}{\dot{\omega }}_{i}+I_{\xi }{\dot{\omega }}_{\xi B}+I_{iB}{\dot{\omega }}_{\xi B} \end{array}\right]\;+\\ & +\;\left[\begin{array}{c} 2M_{t}\omega_{sc}\times {\dot{r}}_{\xi B}\\ {\dot{I}}_{\xi }\omega_{\xi B}+{\dot{I}}_{iB}\omega_{\xi B}+2M_{t}r_{\xi B}\times \left(\omega_{sc}\times {\dot{r}}_{\xi B}\right) \end{array}\right], \end{array}$$ (22)

which can be written in a compact form as follows:

$$\left.\begin{array}{cc} f_{sc}=\left[\begin{array}{c} F_{sc}\\ \tau_{sc} \end{array}\right] & =\left[\begin{array}{cc} D_{v} & D_{v\omega }\\ D_{\omega v} & D_{\omega } \end{array}\right]\left[\begin{array}{c} {\ddot{r}}_{T}\\ {\dot{\omega }}_{sc} \end{array}\right]+\left[\begin{array}{cc} 0 & C_{v}\\ 0 & C_{\omega } \end{array}\right]\left[\begin{array}{c} {\dot{r}}_{T}\\ \omega_{sc} \end{array}\right]+D_{\xi B}+C_{\xi B}\\ & =D_{sc}\ddot{X}+C_{sc}\dot{X}+D_{\xi B}+C_{\xi B} \end{array}\right\},$$ (23)

where $D_{v}\in R^{3\times 3}$ , $D_{v\omega }\in R^{3\times 3}$ , $D_{\omega v}\in R^{3\times 3}$ and $D_{\omega }\in R^{3\times 3}$ are mass submatrices related the linear and angular motion of the spacecraft base, as well as the interaction between the linear and angular motion. Vectors $C_{v}\in R^{3\times 3}$ and $C_{v\omega }\in R^{3\times 3}$ involve the Coriolis and centrifugal terms originating from the motion of the spacecraft base, and vectors ***D***_***ξB***_ and ***C***_***ξB***_ are related to the motion of the CoM *ξ*. The compacted matrix $D_{sc}\in R^{6\times 6}$ is related to the linear and angular motion of the spacecraft base, and the compacted vector $C_{sc}\in R^{6\times 6}$ involves the Coriolis and centrifugal forces.

## 4 Manipulator Dynamics

The mathematical model for the dynamics of a terrestrial robotic arm usually involves both the kinetic and the potential energy (Spong and Vidyasagar, 2008). However, space robots operate in a microgravity environment. For this reason, the terms related to the potential energy in the equation of motion are ignored. Hence, only the kinetic energy was used in the Lagrange–Euler method to derive the equation of motion of the robotic arm of the CFSR.

The Lagrange–Euler equation for an *n* DoF robotic arm is (Spong and Vidyasagar, 2008):

$$\begin{array}{ccc} \tau_{i}=\frac{d}{dt}\left[\frac{\partial L}{\partial {\dot{\theta }}_{i}}\right]-\frac{\partial L}{\partial \theta_{i}} & \text{with} & i=1,\ldots ,n, \end{array}$$ (24)

where *τ*_*i*_ is the torque applied the *i*th joint; *L* is the Lagrangian function of system *L* = *KE*, where *KE* is the kinetic energy of the arm; *θ*_*i*_ represents the *i*th joint angle; and ${\dot{\theta }}_{i}$ is the velocity of the *i*th joint.

### 4.1 Kinetic Energy of an *n* DoF Manipulator

All moving objects have a kinetic energy, and this energy varies according to the mass of the object and the rate of change of the motion (velocity). For a multilink robotic arm, the velocity of each link is represented by the Jacobian matrix and the joint velocity $\dot{\theta }$ . The expression for the linear and angular velocities for the *i*th joint is expressed as follows (Spong and Vidyasagar, 2008):

$$\begin{array}{ccc} v_{i}=J_{v_{mi}}\dot{\theta }, & & \omega_{i}=J_{\omega_{mi}}\dot{\theta }, \end{array},$$ (25)

where $J_{v_{mi}}\in R^{3\times n}$ and $J_{\omega_{mi}}\in R^{3\times n}$ are, respectively, the linear and angular terms of the 6 × *n* Jacobian matrix of the *i*th CoM of the arm, and $R_{L_{i}}\in R^{3\times 3}$ is the rotation matrix of the *i*th link with respect to *∑*_*T*_.

The general equation for the overall kinetic energy for an *n* DoF manipulator is:

$$KE=\frac{1}{2}\overset{n}{\underset{i=1}{\sum }}\left[m_{i}v_{i}^{\prime }v_{i}+\omega_{i}^{\prime }I_{i}\omega_{i}\right],$$ (26)

where **I**_**i**_ is the inertia tensor of the *i*th link about a frame attached at the CoM of the *i*th link. In order to express the inertia tensor in *∑*_*T*_, one has to perform a transformation using the rotation matrix $R_{L_{i}}$ : $I_{i}^{T}=R_{L_{i}}I_{i}R_{L_{i}}^{\prime }$ . Hence, by substituting Eq. 25 into Eq. 26, the expression for the arm’s total kinetic energy is:

$$KE=\frac{1}{2}\dot{\theta }\prime \overset{n}{\underset{i=1}{\sum }}\left[m_{i}J_{v_{i}}^{\prime }J_{v_{i}}+J_{\omega_{i}}^{\prime }R_{L_{i}}I_{i}R_{L_{i}}^{\prime }J_{\omega_{i}}\right]\dot{\theta }.$$ (27)

Eq. 27 can be written in matrix form:

$$KE=\frac{1}{2}{\dot{\theta }}^{\prime }D_{m}\dot{\theta },$$ (28)

where $D_{m}\in R^{n\times n}$ is the symmetric positive definite mass matrix of the robotic manipulator, and it is expressed as:

$$D_{m}=\overset{n}{\underset{i=1}{\sum }}\left(m_{i}J_{v_{i}}^{\prime }J_{v_{i}}+J_{\omega_{i}}^{\prime }R_{L_{i}}I_{i}R_{L_{i}}^{\prime }J_{\omega_{i}}\right).$$ (29)

### 4.2 The Coriolis and Centrifugal Forces

The joints’ rotational motion results in extra forces known as the Coriolis and centrifugal forces. These forces are computed as follows (Spong and Vidyasagar, 2008):

$$c_{kj}=\overset{n}{\underset{i=1}{\sum }}c_{ijk}\dot{\theta_{i}}=\overset{n}{\underset{i=1}{\sum }}\frac{1}{2}\left\{\frac{\partial d_{kj}}{\partial \theta_{j}}+\frac{\partial d_{ki}}{\partial \theta_{j}}+\frac{\partial d_{ij}}{\partial \theta_{k}}\right\}\dot{\theta_{i}},$$ (30)

where *c*_*kj*_ are terms constituting the matrix of Coriolis and centrifugal forces denoted as $C_{m}\in R^{n\times n}$ .

From Eqs 29, 30, the equation describing the linear and angular motion of the arm, the target frame *∑*_*T*_ is:

$$\tau_{m}=D_{m}\ddot{\theta }+C_{m}\dot{\theta }.$$ (31)

## 5 Jacobian Matrix for the Kinematics of the CFSR

The Jacobian matrix relates the velocity of the end-effector, in the Cartesian space, with the velocity of the space robot, in the configuration space. It describes the kinematics of multibody chain, such as the space robot, at the velocity level. It is expressed as:

$$\left[\begin{array}{c} {\dot{r}}_{e}\\ \omega_{e} \end{array}\right]=\left[\begin{array}{cc} J_{sc} & J_{m} \end{array}\right]\left[\begin{array}{c} \dot{X}\\ \dot{\theta } \end{array}\right].$$ (32)

The submatrices constituting the Jacobian matrix are defined as follows:

$$\begin{array}{ccc} J_{sc}={\left[\begin{array}{cc} J_{v_{sc}} & J_{\omega_{sc}} \end{array}\right]}^{\prime } & , & J_{m}={\left[\begin{array}{cc} J_{v_{m}} & J_{\omega_{m}} \end{array}\right]}^{\prime }, \end{array}$$

where $J_{v_{sc}}\in R^{3\times 6}$ and $J_{\omega_{sc}}\in R^{3\times 6}$ are, respectively, the linear and rotational components of the Jacobian matrix for the spacecraft base; and $J_{v_{m}}\in R^{3\times n}$ and $J_{\omega_{m}}\in R^{3\times n}$ are, respectively, the linear and rotational components of the Jacobian matrix for the arm.

The kinematics of a spacecraft can be expressed using either Euler angles or quaternions. Depending on which of these two methods is selected to describe the motion of the space robot, the derivation of the Jacobian matrix varies accordingly. Moreover, when designing a path for the end-effector, the Jacobian matrix related to the end-effector’s position is required, whereas the Jacobian involved in the dynamics is related to the position of the *i*th CoM of the chain. In the following section, the Jacobian matrix of the CFSR is derived for a potential use with both Euler and quaternions. Also, the end-effector’s velocity, as well as the *i*th CoM velocity, is described in *∑*_*T*_.

### 5.1 Jacobian Matrix of the End-Effector in *∑*_*T*_ Using Euler Rate

Deriving the Jacobian matrix of the space robot for use with Euler rate involves several partial derivatives of the rotation matrices as well the spacecraft base’s transformation matrix **R**_***ω***_ defined as follows (Wie, 2008):

$$R_{\omega }=\left[\begin{array}{ccc} 1 & 0 & -sin\left(\beta \right)\\ 0 & cos\left(\alpha \right) & sin\left(\alpha \right)cos\left(\beta \right)\\ 0 & -sin\left(\alpha \right) & cos\left(\alpha \right)cos\left(\beta \right) \end{array}\right]$$ (33)

#### 5.1.1 The Linear Jacobian of the End-Effector

**FIGURE 3:**
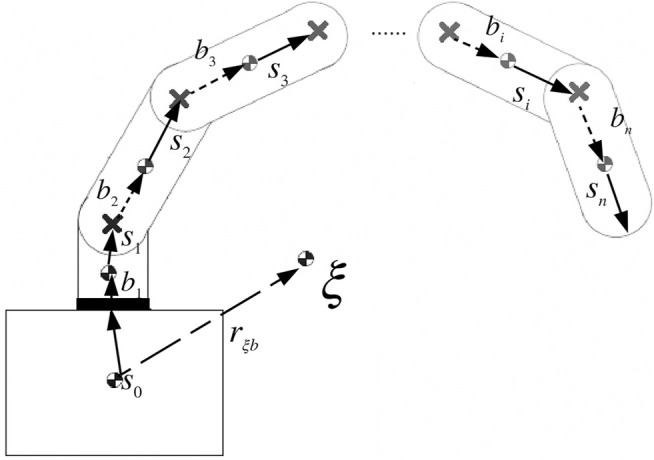
Vector representation for the space robot’s physical parameters (Seddaoui, 2020).

The linear velocity of the end-effector, in the Cartesian space with respect to *∑*_*T*_, is derived using Eq. 2a as follows:

$${\dot{r}}_{e}={\dot{r}}_{T}+{\dot{r}}_{eB}+\omega_{sc}\times r_{eB},$$ (34)

where $r_{eB}\in R^{3}$ is the vector from the origin of *∑*_*B*_ to the end-effector, as seen in Figure 3 and described as:

$$\begin{array}{ll} r_{eB}= & R_{sc}s_{0}+\overset{n}{\underset{i=1}{\sum }}R_{L_{i-1}}l_{i-1}, \end{array}$$ (35a)

$$\begin{array}{ll} {\dot{r}}_{eB}= & {\dot{R}}_{sc}s_{0}+\overset{n}{\underset{i=1}{\sum }}{\dot{R}}_{L_{i-1}}l_{i-1}, \end{array}$$ (35b)

where $s_{0}\in R^{3}$ is the vector from the origin of *∑*_*B*_ to the first joint of the arm. and $l_{i}\in R^{3}$ is the vector from the *i*th joint to the (*i* + 1)^*th*^ joint.

The steps of the differentiation of a rotation matrix were presented by Umetani and Yoshida (1989) as follows:

$$\begin{array}{ll} {\dot{R}}_{sc}= & \frac{\partial R_{sc}}{\partial \alpha }\dot{\alpha }+\frac{\partial R_{sc}}{\partial \beta }\dot{\beta }+\frac{\partial R_{sc}}{\partial \gamma }\dot{\gamma }=\frac{\partial R_{sc}}{\partial \phi }\dot{\phi }. \end{array}$$ (36a)

$$\begin{array}{ll} {\dot{R}}_{L_{i-1}}= & \overset{i-1}{\underset{k=0}{\sum }}\frac{\partial R_{L_{i-1}}}{\partial q_{k}}{\dot{q}}_{k}=\frac{\partial R_{L_{i-1}}}{\partial \phi }\dot{\phi }+\overset{i-1}{\underset{k=1}{\sum }}\frac{\partial R_{L_{i-1}}}{\partial \theta_{k}}{\dot{\theta }}_{k}. \end{array}$$ (36b)

where alpha is the roll angle, beta is pitch angle, and gamma is the yaw angle of the spacecraft base. In addition, phi is the vector of spacecraft base orientation in the target frame.

Using Eq. 36, the linear velocity of the end-effector described by Eq. 34 becomes:

$${\dot{r}}_{e}={\dot{r}}_{T}+\left[\frac{\partial R_{sc}}{\partial \phi }s_{0}+\overset{n}{\underset{i=1}{\sum }}\frac{\partial R_{L_{i-1}}}{\partial \phi }l_{i-1}\right]\dot{\phi }+\left[\overset{n}{\underset{i=1}{\sum }}\overset{i-1}{\underset{k=1}{\sum }}\frac{\partial R_{L_{i-1}}}{\partial \theta_{k}}l_{i-1}\right]\dot{\theta }-{\left[r_{eB}\right]}_{\times }\omega_{sc}.$$ (37)

The last term of Eq. 37 involves the body rate ***ω***_***sc***_ which can be a function of Euler rate and **R**_***ω***_ using Eq. 33. The linear velocity described by Eq. 37 then becomes:

$${\dot{r}}_{e}={\dot{r}}_{T}+\left[\frac{\partial R_{sc}}{\partial \phi }s_{0}+\overset{n}{\underset{i=1}{\sum }}\frac{\partial R_{L_{i-1}}}{\partial \phi }l_{i-1}\right]\dot{\phi }+\left[\overset{n}{\underset{i=1}{\sum }}\overset{i-1}{\underset{k=1}{\sum }}\frac{\partial R_{L_{i-1}}}{\partial \theta_{k}}l_{i-1}\right]\dot{\theta }-{\left[r_{eB}\right]}_{\times }R_{\omega }\dot{\phi }.$$ (38)

From Eq. 38, the linear Jacobian submatrices for both the spacecraft base and the arm are:

$$\left.\begin{array}{ccc} J_{v_{sc}} & =\left[\begin{array}{cccc} E & J_{v_{sc}}^{\prime \prime }\left(1\right) & J_{v_{sc}}^{\prime \prime }\left(2\right) & J_{v_{sc}}^{\prime \prime }\left(3\right) \end{array}\right]\\ & J_{v_{sc}}^{\prime \prime }\left(1\right) & =\frac{\partial R_{sc}}{\partial \alpha }s_{0}+\sum_{i=1}^{n}\frac{\partial R_{L_{i-1}}}{\partial \alpha }l_{i-1}-{\left[r_{eB}\right]}_{\times }R_{\omega }e_{1},\\ & J_{v_{sc}}^{\prime \prime }\left(2\right) & =\frac{\partial R_{sc}}{\partial \beta }s_{0}+\sum_{i=1}^{n}\frac{\partial R_{L_{i-1}}}{\partial \beta }l_{i-1}-{\left[r_{eB}\right]}_{\times }R_{\omega }e_{2},\\ & J_{v_{sc}}^{\prime \prime }\left(3\right) & =\frac{\partial R_{sc}}{\partial \gamma }s_{0}+\sum_{i=1}^{n}\frac{\partial R_{L_{i-1}}}{\partial \gamma }l_{i-1}-{\left[r_{eB}\right]}_{\times }R_{\omega }e_{3},\\ J_{v_{m}} & =\left[\begin{array}{cccc} J_{v_{m}}^{\prime \prime }\left(1\right) & J_{v_{m}}^{\prime \prime }\left(2\right) & \ldots & J_{v_{m}}^{\prime \prime }\left(n\right) \end{array}\right]\\ & J_{v_{m}}^{\prime \prime }\left(j\right)= & \sum_{i=1}^{j}\sum_{k=1}^{i-1}\frac{\partial R_{L_{i-1}}}{\partial \theta_{k}}l_{i-1},\;j=1,2,\ldots ,n \end{array}\right\},$$ (39)

where $E\in R^{3\times 3}$ , $J_{v_{sc}}^{\prime \prime }\left(1\right),J_{v_{sc}}^{\prime \prime }\left(2\right),J_{v_{sc}}^{\prime \prime }\left(3\right)\in R^{3}$ , $J_{v_{m}}^{\prime \prime }\left(j\right)\in R^{3}$ and ***e***_**1**_, ***e***_**2**_, ***e***_**3**_ are unit vectors.

#### 5.1.2 The Rotational Jacobian of the End-Effector

The angular velocity of the end-effector can be expressed as:

$$\left.\begin{array}{ccc} \omega_{e} & = & \omega_{sc}+\omega_{eB}\\ & \omega_{eB} & =\sum_{i=1}^{n}R_{L_{i}}{\left[\begin{array}{ccc} 0 & 0 & \dot{\theta_{i}} \end{array}\right]}^{\prime } \end{array}\right\},$$ (40)

where $\omega_{e}\in R^{3}$ is the angular velocity of the end-effector in *∑*_*T*_, $\omega_{eB}\in R^{3}$ is the angular velocity of the end-effector in *∑*_*B*_, and $\omega_{sc}\in R^{3}$ is the angular velocity of the spacecraft base in *∑*_*T*_.

Using Eqs 33–40, ***ω***_***e***_ becomes:

$$\omega_{e}=R_{\omega }\left[\begin{array}{c} \dot{\alpha }\\ \dot{\beta }\\ \dot{\gamma } \end{array}\right]+\overset{n}{\underset{i=1}{\sum }}R_{L_{i}}\left[\begin{array}{c} 0\\ 0\\ \dot{\theta_{i}} \end{array}\right].$$ (41)

From Eq. 41, the rotational Jacobian submatrices for both the spacecraft base and the arm are:

$$\left.\begin{array}{ccc} J_{\omega_{sc}} & =\left[\begin{array}{cccc} 0 & J_{\omega_{sc}}^{\prime \prime }\left(1\right) & J_{\omega_{sc}}^{\prime \prime }\left(2\right) & J_{\omega_{sc}}^{\prime \prime }\left(3\right) \end{array}\right]\\ & J_{\omega_{sc}}^{\prime \prime }\left(1\right) & =R_{\omega }e_{1}\\ & J_{\omega_{sc}}^{\prime \prime }\left(2\right) & =R_{\omega }e_{2}\\ & J_{\omega_{sc}}^{\prime \prime }\left(3\right) & =R_{\omega }e_{3}\\ J_{\omega_{m}} & =\left[\begin{array}{cccc} J_{\omega_{m}}^{\prime \prime }\left(1\right) & J_{\omega_{m}}^{\prime \prime }\left(2\right) & \ldots & J_{\omega_{m}}^{\prime \prime }\left(n\right) \end{array}\right]\\ & J_{\omega_{m}}^{\prime \prime }\left(j\right)= & \sum_{i=0}^{j}R_{L_{i}}e_{3}\;j=1,2,\ldots ,n \end{array}\right\},$$ (42)

where $0\in R^{3\times 3}\;J_{\omega_{sc}}^{\prime \prime }\left(j\right)\in R^{3}$ and $J_{\omega_{m}}^{\prime \prime }\left(j\right)\in R^{3}$ and ***e***_**1**_, ***e***_**2**_ and ***e***_**3**_ are unit vectors.

#### 5.1.3 The Full Jacobian Translating the Velocities of the End-Effector

From Eqs 39–42, the overall Jacobian matrix for the space robot is as follows:

$$\left[\begin{array}{cc} J_{sc} & J_{m} \end{array}\right]=\left[\begin{array}{cccc} E & J_{v_{sc}}^{\prime \prime } & | & J_{v_{m}}\\ 0 & J_{\omega_{sc}}^{\prime \prime } & | & J_{\omega_{m}} \end{array}\right].$$ (43)

### 5.2 Jacobian Matrix of the *i*th CoM in *∑*_*T*_ Using Euler Rate

The Jacobian described by Eq. 43 is used to find the position of the end-effector in the Cartesian space, given a configuration or the inverse procedure for path planning. However, the matrices $J_{v_{m_{i}}}$ and $J_{\omega_{m_{i}}}$ of Eq. 29 relate to the velocity of the CoM of the *i*th link with the velocities in the configuration space. For this reason, the equations for $J_{v_{m_{i}}}$ and $J_{\omega_{m_{i}}}$ in Eq. 29 are different from those for $J_{v_{m}}$ and $J_{\omega_{m}}$ in Eqs 39–42.

#### 5.2.1 The Linear Jacobian of the *i*th CoM

Using Eq. 2a, the linear velocity of the *i*th CoM, with respect to *∑*_*T*_, is determined as follows:

$${\dot{r}}_{i}={\dot{r}}_{T}+{\dot{r}}_{iB}+\omega_{sc}\times r_{iB},$$ (44)

where ***r***_***iB***_ is the vector from the origin of *∑*_*B*_ to the *i*th CoM, as seen in Figure 3. It is represented by the following equation:

$$r_{iB}=\overset{i}{\underset{j=1}{\sum }}\left(R_{L_{j}}b_{j}+R_{L_{j-1}}s_{j-1}\right),$$ (45)

where $b_{i}\in R^{3}$ is the vector from the *i*th joint to the CoM of the *i*th link, and $s_{i-1}\in R^{3}$ is the vector from the CoM of the (*i* − 1)^*th*^ link to the *i*th joint as shown in Figure 3.

Differentiating Eq. 45 and using the derivatives of the rotation matrix in Eq. 36 give:

$${\dot{r}}_{iB}=\overset{i}{\underset{j=1}{\sum }}\left[\frac{\partial R_{L_{j}}}{\partial \phi }b_{j}+\frac{\partial R_{L_{j-1}}}{\partial \phi }s_{j-1}\right]\dot{\phi }+\overset{i}{\underset{j=1}{\sum }}\left[\overset{i}{\underset{k=1}{\sum }}\frac{\partial R_{L_{j}}}{\partial \theta_{k}}b_{j}+\overset{j-1}{\underset{k=1}{\sum }}\frac{\partial R_{L_{j-1}}}{\partial \theta_{k}}s_{j-1}\right]\dot{\theta }.$$ (46)

By substituting Eq. 46 into Eq. 44, the equation for the linear velocity of the *i*th CoM becomes:

$$\begin{array}{cc} {\dot{r}}_{i} & ={\dot{r}}_{T}+\sum_{j=1}^{i}\left[\frac{\partial R_{L_{j}}}{\partial \phi }b_{j}+\frac{\partial R_{L_{j-1}}}{\partial \phi }s_{j-1}\right]\dot{\phi }\\ & +\sum_{j=1}^{i}\left[\sum_{k=1}^{j}\frac{\partial R_{L_{j}}}{\partial \theta_{k}}b_{j}+\sum_{k=1}^{j-1}\frac{\partial R_{L_{j-1}}}{\partial \theta_{k}}s_{j-1}\right]\dot{\theta }-{\left[r_{iB}\right]}_{\times }R_{\omega }\dot{\phi }. \end{array}$$ (47)

From Eq. 47, the linear Jacobian matrices, derived for the dynamics, for both the base spacecraft and the arm are:

$$\left.\begin{array}{ccc} J_{v_{{sc}_{i}}} & = & \left[\begin{array}{cccc} E & J_{v_{{sc}_{i}}}^{\prime \prime }\left(1\right) & J_{v_{sc}}^{\prime \prime }\left(2\right) & J_{v_{sc}}^{\prime \prime }\left(3\right) \end{array}\right]\\ & J_{v_{{sc}_{i}}}^{\prime \prime }\left(1\right) & =\sum_{j=1}^{i}\left[\frac{\partial R_{L_{j}}}{\partial \alpha }b_{j}+\frac{\partial R_{L_{j-1}}}{\partial \alpha }s_{j-1}\right]-{\left[r_{iB}\right]}_{\times }R_{\omega }e_{1}\\ & J_{v_{{sc}_{i}}}^{\prime \prime }\left(2\right) & =\sum_{j=1}^{i}\left[\frac{\partial R_{L_{j}}}{\partial \beta }b_{j}+\frac{\partial R_{L_{j-1}}}{\partial \beta }s_{j-1}\right]-{\left[r_{iB}\right]}_{\times }R_{\omega }e_{2}\\ & J_{v_{{sc}_{i}}}^{\prime \prime }\left(3\right) & =\sum_{j=1}^{i}\left[\frac{\partial R_{L_{j}}}{\partial \gamma }b_{j}+\frac{\partial R_{L_{j-1}}}{\partial \gamma }s_{j-1}\right]-{\left[r_{iB}\right]}_{\times }R_{\omega }e_{3}\\ J_{v_{m_{i}}}\left(j\right) & = & \sum_{j=0}^{i}\left[\overset{j}{\underset{k=1}{\sum }}\frac{\partial R_{L_{j}}}{\partial \theta_{k}}b_{j}+\sum_{k=1}^{j-1}\frac{\partial R_{L_{j-1}}}{\partial \theta_{k}}s_{j-1}\right] \end{array}\right\},$$

where $J_{v_{{sc}_{i}}}$ is the *i*th linear Jacobian submatrix of the spacecraft base resulting from the motion of the base itself and the arm, and $J_{v_{m_{i}}}\left(j\right)$ is the *j*th column of the linear Jacobian matrix of the *i*th CoM of the arm.

#### 5.2.2 The Rotational Jacobian of the *i*th CoM

The angular velocity of the *i*th CoM in *∑*_*T*_ is expressed as follows:

$$\left.\begin{array}{ccc} \omega_{e} & = & \omega_{sc}+\omega_{i}^{sc}\\ & \omega_{i}^{sc} & =\sum_{j=1}^{i}R_{L_{j}}{\left[\begin{array}{ccc} 0 & 0 & \dot{\theta_{i}} \end{array}\right]}^{\prime } \end{array}\right\},$$ (48)

where $\omega_{i}^{sc}\in R^{3}$ is the angular velocity of the *i*th CoM in *∑*_*B*_. Based on Eq. 42, the rotational Jacobian for the *i*th CoM is:

$$\left.\begin{array}{ccc} J_{\omega_{sc}} & =\left[\begin{array}{cccc} 0 & J_{\omega_{sc}}^{\prime \prime }\left(1\right) & J_{\omega_{sc}}^{\prime \prime }\left(2\right) & J_{\omega_{sc}}^{\prime \prime }\left(3\right) \end{array}\right]\\ & J_{\omega_{sc}}^{\prime \prime }\left(1\right) & =R_{\omega }e_{1}\\ & J_{\omega_{sc}}^{\prime \prime }\left(2\right) & =R_{\omega }e_{2}\\ & J_{\omega_{sc}}^{\prime \prime }\left(3\right) & =R_{\omega }e_{3}\\ J_{\omega_{m_{i}}}\left(j\right) & = & \sum_{j=1}^{i}R_{L_{j}}e_{3} \end{array}\right\},$$ (49)

where $J_{\omega_{m_{i}}}\left(j\right)$ is the *j*th column of the rotational Jacobian matrix of the *i*th CoM of the manipulator.

### 5.3 Jacobian Matrix of the End-Effector in *∑*_*T*_ Using Body Rate

It is known that singularities can occur when using the Euler rate $\dot{\phi }$ to express the attitude of the spacecraft base. Hence, quaternions are preferred to avoid singularities. For this reason, the Jacobian matrix has to be expressed with respect to the body rate ***ω***_***sc***_ to allow an easy transformation to quaternions. In this case, there are no partial derivatives of the rotation matrices involved.

#### 5.3.1 The Linear Jacobian of the End-Effector Using Body Rate

The linear velocity of the end-effector, described by Eq. 34, is here derived in a different manner to maintain the body rate term in the equation. This is performed using another method for the derivative of the rotation matrices **R**_**sc**_ and $R_{L_{i}}$ , hereafter presented as (Spong and Vidyasagar, 2008):

$$\left.\begin{array}{cc} {\dot{R}}_{sc} & ={\left[\omega_{sc}\right]}_{\times }R_{sc}\\ & ={\left[e_{1}\right]}_{\times }R_{sc}\omega_{sc_{x}}+{\left[e_{2}\right]}_{\times }R_{sc}\omega_{sc_{y}}+{\left[e_{3}\right]}_{\times }R_{sc}\omega_{sc_{z}} \end{array}\right\},$$ (50)

and

$$\left.\begin{array}{cc} {\dot{R}}_{L_{i-1}} & =\sum_{j=1}^{i-1}R_{L_{j-1}}{\left[\omega_{j}\right]}_{\times }R_{L_{i-1}}\\ & ={\left[\omega_{sc}\right]}_{\times }R_{L_{i-1}}l_{i-1}+\sum_{j=1}^{i-1}R_{L_{j-1}}{\left[\omega_{j}\right]}_{\times }R_{L_{i-1}}\\ & ={\left[e_{1}\right]}_{\times }R_{L_{i-1}}l_{i-1}\omega_{sc_{x}}+{\left[e_{2}\right]}_{\times }R_{L_{i-1}}l_{i-1}\omega_{sc_{y}}+{\left[e_{3}\right]}_{\times }R_{L_{i-1}}l_{i-1}\omega_{sc_{z}}\;+\\ & \;+\;\sum_{j=1}^{i-1}R_{L_{j-1}}{\left[e_{3}\right]}_{\times }R_{L_{i-1}}{\dot{\theta }}_{j} \end{array}\right\}.$$ (51)

Substituting Eqs 50, 51 into Eq. 34 gives:

$$\left.\begin{array}{ccc} {\dot{r}}_{e} & ={\dot{r}}_{T}+{\left[\omega_{sc}\right]}_{\times }R_{sc}s_{0}+\sum_{i=1}^{n}{\left[\omega_{sc}\right]}_{\times }R_{L_{i-1}}l_{i-1}-{\left[r_{eB}\right]}_{\times }\omega_{sc},\\ & ={\dot{r}}_{T}+\left({\left[e_{1}\right]}_{\times }R_{sc}s_{0}+\sum_{i=1}^{n}{\left[e_{1}\right]}_{\times }R_{L_{i-1}}l_{i-1}-{\left[r_{eB}\right]}_{\times }e_{1}\right)\omega_{sc_{x}}\\ & & +\left({\left[e_{2}\right]}_{\times }R_{sc}s_{0}+\sum_{i=1}^{n}{\left[e_{2}\right]}_{\times }R_{L_{i-1}}l_{i-1}-{\left[r_{eB}\right]}_{\times }e_{2}\right)\omega_{sc_{y}}\\ & +\left({\left[e_{3}\right]}_{\times }R_{sc}s_{0}+\sum_{i=1}^{n}{\left[e_{3}\right]}_{\times }R_{L_{i-1}}l_{i-1}-{\left[r_{eB}\right]}_{\times }e_{3}\right)\omega_{sc_{z}}\\ & +\sum_{i=1}^{n}\sum_{j=1}^{i-1}R_{L_{j-1}}{\left[e_{3}\right]}_{\times }R_{L_{i-1}}l_{i-1}\dot{\theta_{j}} \end{array}\right\}.$$ (52)

Expressing Eq. 52 in matrix form gives the following Jacobian submatrices for both the spacecraft base and the arm:

$$\left.\begin{array}{ccc} J_{v_{sc}} & =\left[\begin{array}{cccc} E & J_{v_{sc}}^{\prime \prime }\left(1\right) & J_{v_{sc}}^{\prime \prime }\left(2\right) & J_{v_{sc}}^{\prime \prime }\left(3\right) \end{array}\right]\\ & J_{v_{sc}}^{\prime \prime }\left(1\right) & ={\left[e_{1}\right]}_{\times }R_{sc}s_{0}+\sum_{i=1}^{n}{\left[e_{1}\right]}_{\times }R_{L_{i-1}}l_{i-1}-{\left[r_{eB}\right]}_{\times }e_{1},\\ & J_{v_{sc}}^{\prime \prime }\left(2\right) & ={\left[e_{2}\right]}_{\times }R_{sc}s_{0}+\sum_{i=1}^{n}{\left[e_{2}\right]}_{\times }R_{L_{i-1}}l_{i-1}-{\left[r_{eB}\right]}_{\times }e_{2},\\ & J_{v_{sc}}^{\prime \prime }\left(3\right) & ={\left[e_{3}\right]}_{\times }R_{sc}s_{0}+\sum_{i=1}^{n}{\left[e_{3}\right]}_{\times }R_{L_{i-1}}l_{i-1}-{\left[r_{eB}\right]}_{\times }e_{3},\\ J_{v_{m}} & =\left[\begin{array}{cccc} J_{v_{m}}^{\prime \prime }\left(1\right) & J_{v_{m}}^{\prime \prime }\left(2\right) & \ldots & J_{v_{m}}^{\prime \prime }\left(n\right) \end{array}\right]\\ & J_{v_{m}}^{\prime \prime }\left(j\right)= & \sum_{k=1}^{j}\sum_{i=1}^{i-1}R_{L_{k-1}}{\left[e_{3}\right]}_{\times }R_{L_{i-1}}l_{i-1},\;j=1,2,\ldots ,n \end{array}\right\}.$$ (53)

#### 5.3.2 The Rotational Jacobian of the End-Effector Using Body Rate

Using Eq. 48, the rotational Jacobian submatrix, used to find the angular velocity of the end-effector, with respect to body rate expressed in matrix form is:

$$\left.\begin{array}{ccc} J_{\omega_{sc}} & =\left[\begin{array}{cccc} 0 & e_{1} & e_{2} & e_{3} \end{array}\right]\\ J_{\omega_{m}} & =\left[\begin{array}{cccc} J_{\omega_{m}}^{\prime \prime }\left(1\right) & J_{\omega_{m}}^{\prime \prime }\left(2\right) & \ldots & J_{\omega_{m}}^{\prime \prime }\left(n\right) \end{array}\right]\\ & J_{\omega_{m}}^{\prime \prime }\left(j\right)= & \sum_{i=1}^{j}R_{L_{i}}e_{3}\;j=1,2,\ldots ,n \end{array}\right\},$$ (54)

where $0\in R^{3\times 3}$ and ***e***_**1**_, ***e***_**2**_, ***e***_**3**_ are unit vectors.

### 5.4 Jacobian Matrix of the *i*th CoM in *∑*_*T*_ Using Body Rate

Similar to the Jacobian matrices of the links’ CoMs using Euler rate, deriving these matrices using body rate is necessary when quaternions are utilized.

#### 5.4.1 The Linear Jacobian of the *i*th CoM Using Body Rate

Differentiating Eq. 45 using the derivatives of the rotation matrices in Eqs 50, 51 gives:

$$\begin{array}{cc} {\dot{r}}_{iB} & =\sum_{j=1}^{i}\left({\left[\omega_{sc}\right]}_{\times }R_{L_{j}}b_{j}+{\left[\omega_{sc}\right]}_{\times }R_{L_{j-1}}s_{j-1}\right)\;\\ & \;+\;\sum_{j=1}^{i}\left(\sum_{k=1}^{j}R_{L_{k-1}}{\left[\omega_{k}\right]}_{\times }R_{L_{j}}b_{j}+\sum_{k=1}^{j-1}R_{L_{k-1}}{\left[\omega_{k}\right]}_{\times }R_{L_{j-1}}s_{j-1}\right). \end{array}$$ (55)

Substituting Eq. 55 into Eq. 44 gives the expression for the linear velocity of the *i*th link using the spacecraft body rate as follows:

$$\begin{array}{cc} {\dot{r}}_{i} & ={\dot{r}}_{T}+\left(\sum_{j=1}^{i}\left({\left[e_{1}\right]}_{\times }R_{L_{j}}b_{j}+{\left[e_{1}\right]}_{\times }R_{L_{j-1}}s_{j-1}\right)-{\left[r_{iB}\right]}_{\times }e_{1}\right)\omega_{x}\;\\ & \;+\;\left(\sum_{j=1}^{i}\left({\left[e_{2}\right]}_{\times }R_{L_{j}}b_{j}+{\left[e_{2}\right]}_{\times }R_{L_{j-1}}s_{j-1}\right)-{\left[r_{iB}\right]}_{\times }e_{2}\right)\omega_{y}\;\\ & \;+\;\left(\sum_{j=1}^{i}\left({\left[e_{3}\right]}_{\times }R_{L_{j}}b_{j}+{\left[e_{3}\right]}_{\times }R_{L_{j-1}}s_{j-1}\right)-{\left[r_{iB}\right]}_{\times }e_{3}\right)\omega_{z}\;\\ & +\;\overset{i}{\underset{j=1}{\sum }}\left(\sum_{k=1}^{j}R_{L_{k-1}}{\left[e_{3}\right]}_{\times }R_{L_{j}}b_{j}+\sum_{k=1}^{j-1}R_{L_{k-1}}{\left[e_{3}\right]}_{\times }R_{L_{j-1}}s_{j-1}\right)\dot{\theta }. \end{array}$$ (56)

Expressing Eq. 56 in matrix form gives the following linear Jacobian submatrices to compute the linear velocity of the *i*th link:

$$\left.\begin{array}{ccc} J_{v_{{sc}_{i}}} & =\left[\begin{array}{cccc} E & J_{v_{{sc}_{i}}}^{\prime \prime }\left(1\right) & J_{v_{{sc}_{i}}}^{\prime \prime }\left(2\right) & J_{v_{{sc}_{i}}}^{\prime \prime }\left(3\right) \end{array}\right]\\ & J_{v_{{sc}_{i}}}^{\prime \prime }\left(1\right) & =\sum_{j=1}^{i}\left({\left[e_{1}\right]}_{\times }R_{L_{j}}b_{j}+{\left[e_{1}\right]}_{\times }R_{L_{j-1}}s_{j-1}\right)-{\left[r_{iB}\right]}_{\times }e_{1}\\ & J_{v_{{sc}_{i}}}^{\prime \prime }\left(2\right) & =\sum_{j=1}^{i}\left({\left[e_{2}\right]}_{\times }R_{L_{j}}b_{j}+{\left[e_{2}\right]}_{\times }R_{L_{j-1}}s_{j-1}\right)-{\left[r_{iB}\right]}_{\times }e_{2}\\ & J_{v_{{sc}_{i}}}^{\prime \prime }\left(3\right) & =\sum_{j=1}^{i}\left({\left[e_{3}\right]}_{\times }R_{L_{j}}b_{j}+{\left[e_{3}\right]}_{\times }R_{L_{j-1}}s_{j-1}\right)-{\left[r_{iB}\right]}_{\times }e_{3}\\ J_{v_{m_{i}}}\left(j\right) & = & \overset{i}{\underset{j=1}{\sum }}\left(\sum_{k=1}^{j}R_{L_{k-1}}{\left[e_{3}\right]}_{\times }R_{L_{j}}b_{j}+\sum_{k=1}^{j-1}R_{L_{k-1}}{\left[e_{3}\right]}_{\times }R_{L_{j-1}}s_{j-1}\right)\\ & & j=1,2,\ldots ,n \end{array}\right\}.$$ (57)

#### 5.4.2 The Angular Jacobian of the *i*th CoM Using Body Rate

It is similar to the rotational Jacobian in Eq. 49, and it is expressed as follows:

$$\left.\begin{array}{ccc} J_{\omega_{sc}} & = & \left[\begin{array}{cccc} 0 & e_{1} & e_{2} & e_{3} \end{array}\right]\\ & J_{\omega_{m_{i}}}\left(j\right)= & \sum_{j=1}^{i}R_{L_{j}}e_{3}\;j=1,2,\ldots ,n \end{array}\right\}$$ (58)

#### 5.4.3 Simulation Validation of the Kinematic Equations

It is important to validate the kinematic model of the space robot before integrating it with the equations of the dynamics. As presented in this article, the equations for the motion of the end-effector and the *i*th CoM are different. The inverse kinematics computation uses Eq. 35a as it represents the end-effector’s motion. The motion of the links’ CoM, represented by Eq. 44, is used to compute the robot’s dynamics. The simulation-based validation uses these two equations to define the motion of the manipulator. Testing these two equations for different space robot configurations will help validate the kinematics equations by verifying if, for each configuration, the position of the *i*th CoM corresponds to the *i*th link.

For the simulations, the joint angles were computed separately from the positions of the end-effector and the links’ CoM and were plotted in the same plot to check if they coincide. The check was performed not only visually but also by comparing the positions of each CoM with respect to its link, in six arbitrary sets of space robot configurations, including the arm’s joints and spacecraft’s attitude. This process was carried out to validate the accuracy of the two sets of equations before integrating them with the kinematics and dynamics.

**FIGURE 4:**
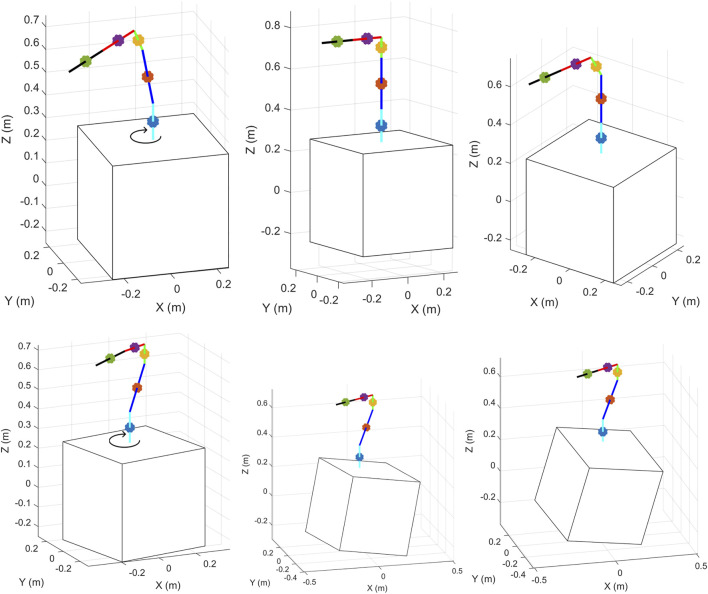
Links CoMs’ positions using different joint angles for arm and base attitude (Seddaoui, 2020).

The results are shown in Figure 4, where the first three configurations have a fixed spacecraft base and joints, respectively, equal to: ***θ*** = [11° 11° 11° 0° 0° 0°]′, ***θ*** = [0° 0° 0° 0° 0° 0°]′, and ***θ*** = [0° 0° 30° 0° 0° 0°]′. The last three configurations have a fixed joint angle equal to ***θ*** = [0°–30° 0° 30° 30° 0°]′ and three different sets of base rotations defined, respectively, as rotation about *z* axis, rotations about *z* and *y* axes, and rotations about *z*, *y* and *x* axes. This simulation-based validation proves the correctness of the equations used in the derivation of the Jacobian matrices that describe the kinematics of the CFSR.

## 6 The Dynamic Coupling Between the Arm and the Spacecraft Base

The dynamic coupling describes the interactions between the arm and its base during the motion of the space robot. The impact of the motion of the base on the arm is introduced through the Jacobian matrix. In other words, desired changes in the pose of the spacecraft base will systematically change the end-effector’s position through the Jacobian matrix. This feature is used to aid the arm to reach the target. However, the motion of the arm produces undesired changes in the pose of the spacecraft base, which in return affect the end-effector’s position. This results from the motion of the system’s CoM, which corresponds to the last two terms of Eq. 22. These two terms are:

$$\begin{array}{ccc} D_{\xi B}+C_{\xi B} & = & \left[\begin{array}{c} M_{t}{\ddot{r}}_{\xi B}\\ M_{t}r_{\xi B}\times {\ddot{r}}_{\xi B}+\sum_{i=1}^{n}I_{i}{\dot{\omega }}_{i}+I_{\xi }{\dot{\omega }}_{\xi B}+I_{iB}{\dot{\omega }}_{\xi B} \end{array}\right]\;+\\ & & +\;\left[\begin{array}{c} 2M_{t}\omega_{sc}\times {\dot{r}}_{\xi B}\\ {\dot{I}}_{\xi }\omega_{\xi B}+{\dot{I}}_{iB}\omega_{\xi B}+2M_{t}r_{\xi B}\times \left(\omega_{sc}\times {\dot{r}}_{\xi B}\right) \end{array}\right]. \end{array}$$ (59)

The angular velocity ***ω***_***ξB***_ is expressed as follows (Curtis, 2013):

$$\omega_{\xi B}=\frac{r_{\xi B}\times {\dot{r}}_{\xi B}}{r_{\xi B}^{2}}.$$ (60)

The derivative of ***ω***_***ξB***_, described by Eg (60), is defined as follows:

$${\dot{\omega }}_{\xi B}=\frac{r_{\xi B}\times {\ddot{r}}_{\xi B}}{r_{\xi B}^{2}}+2\frac{r_{\xi B}\times {\dot{r}}_{\xi B}}{r_{\xi B}^{3}}.$$ (61)

Vector ***r***_***ξB***_ and its first derivative are defined as follows:

$$\begin{array}{ll} r_{\xi B} & =\frac{1}{M_{t}}\overset{n}{\underset{i=1}{\sum }}m_{i}r_{iB}, \end{array}$$ (62a)

$$\begin{array}{ll} {\dot{r}}_{\xi B} & =\frac{1}{M_{t}}\overset{n}{\underset{i=1}{\sum }}m_{i}{\dot{r}}_{iB}. \end{array}$$ (62b)

In order to derive the first term of Eq. 59, the second derivative of vector ***r***_***iB***_ has to be computed. Its first derivative is defined by Eq. 55, which was used to find the linear term of the Jacobian matrix of the *i*th link in *∑*_*T*_. Contrarily, vector ***r***_***iB***_ is here defined in *∑*_*B*_. This changes the origin of the rotation matrices from *∑*_*T*_ to *∑*_*B*_. Hence, the first derivative of vector ***r***_***iB***_ is computed as follows:

$${\dot{r}}_{iB}=\overset{i}{\underset{j=1}{\sum }}\left(\overset{j}{\underset{k=1}{\sum }}R_{L_{k-1}}{\left[\omega_{k}\right]}_{\times }R_{L_{j}}b_{j}+\overset{j-1}{\underset{k=1}{\sum }}R_{L_{k-1}}{\left[\omega_{k}\right]}_{\times }R_{L_{j-1}}s_{j-1}\right).$$ (63)

An inspection of Eq. 63 shows that it is similar to the linear part of the *i*th link’s Jacobian matrix as described in Eq. 57. Therefore, the first derivative of vector ***r***_***iB***_ is given as:

$${\dot{r}}_{iB}=\overset{n}{\underset{i=1}{\sum }}J_{v_{m_{i}}}\dot{\theta }.$$ (64)

The second derivative of vector ***r***_***iB***_ is then defined as:

$${\ddot{r}}_{iB}=\overset{n}{\underset{i=1}{\sum }}\left(J_{v_{m_{i}}}\ddot{\theta }+{\dot{J}}_{v_{m_{i}}}\dot{\theta }\right).$$ (65)

The expression for the first term of Eq. 59 is defined as follows:

$${\ddot{r}}_{\xi B}=\frac{1}{M_{t}}\overset{n}{\underset{i=1}{\sum }}m_{i}J_{v_{m_{i}}}\ddot{\theta }+\frac{1}{M_{t}}\overset{n}{\underset{i=1}{\sum }}m_{i}{\dot{J}}_{v_{m_{i}}}\dot{\theta }.$$ (66)

The term $\sum_{i=1}^{n}I_{i}{\dot{\omega }}_{i}$ is to be further derived to make it function of the joint velocity $\dot{\theta }$ . This is achieved through finding the equation for ***ω***_***i***_ as follows:

$$\omega_{i}=\overset{i}{\underset{j=1}{\sum }}R_{L_{j}}\theta_{j}.$$ (67)

Eq. 67 is similar to the rotational part of the *i*th link’s Jacobian matrix. Hence:

$$\begin{array}{ll} \overset{n}{\underset{i=1}{\sum }}I_{i}\omega_{i} & =\overset{n}{\underset{i=1}{\sum }}I_{i}J_{\omega_{m_{i}}}\dot{\theta } \end{array}$$ (68a)

$$\begin{array}{ll} \overset{n}{\underset{i=1}{\sum }}I_{i}{\dot{\omega }}_{i} & =\overset{n}{\underset{i=1}{\sum }}I_{i}J_{\omega_{m_{i}}}\ddot{\theta }+\overset{n}{\underset{i=1}{\sum }}I_{i}\dot{J_{\omega_{m_{i}}}}\dot{\theta }. \end{array}$$ (68b)

Substituting Eqs 62a, 62b, (Eq. 66), and (Eq. 68b) into (Eq. 59) and rearranging give:

$$D_{\xi B}+C_{\xi B}=\left[\begin{array}{c} \sum_{i=1}^{n}m_{i}J_{v_{m_{i}}}\\ \left(1+\frac{I_{\xi }+I_{iB}}{M_{t}r_{\xi B}^{2}}\right){\left[r_{\xi B}\right]}_{\times }\sum_{i=1}^{n}m_{i}J_{v_{m_{i}}}+\sum_{i=1}^{n}I_{i}J_{\omega_{m_{i}}} \end{array}\right]\ddot{\theta }+\left[\begin{array}{c} {Csc.m}_{v}\\ {Csc.m}_{\omega } \end{array}\right]\dot{\theta },$$ (69)

where matrices **Csc.m**_**v**_ and **Csc.m**_*ω*_ are defined as follows:

$$\begin{array}{cc} {Csc.m}_{v} & =\sum_{i=1}^{n}\left(m_{i}{\dot{J}}_{v_{m_{i}}}+2\omega_{sc}\times J_{v_{m_{i}}}\right),\\ {Csc.m}_{\omega } & =\sum_{i=1}^{n}\left(I_{i}{\dot{J}}_{\omega_{m_{i}}}\;+2r_{\xi B}\times \left(\omega_{sc}\times J_{v_{m_{i}}}\right)\right.\\ & \left.+\;\frac{{\dot{I}}_{\xi }+{\dot{I}}_{iB}}{M_{t}r_{\xi B}^{2}}{\left[r_{\xi B}\right]}_{\times }\sum_{i=1}^{n}m_{i}J_{v_{m_{i}}}+2\frac{I_{\xi }+I_{iB}}{M_{t}r_{\xi B}^{3}}{\left[r_{\xi B}\right]}_{\times }\sum_{i=1}^{n}m_{i}J_{v_{m_{i}}}\right). \end{array}$$

Writing Eq. 69 in a compact matrix form gives:

$$D_{\xi B}+C_{\xi B}=\left[\begin{array}{c} D_{{sc.m}_{v}}\\ D_{{sc.m}_{\omega }} \end{array}\right]\ddot{\theta }+\left[\begin{array}{c} C_{{sc.m}_{v}}\\ C_{{sc.m}_{\omega }} \end{array}\right]\dot{\theta }=D_{sc.m}\ddot{\theta }+C_{sc.m}\dot{\theta },$$ (70)

where $D_{{sc.m}_{v}}\in R^{3\times n}$ and $D_{{sc.m}_{\omega }}\in R^{3\times n}$ are matrices related to the dynamic coupling between the arm and its spacecraft base, and matrices $C_{{sc.m}_{v}}\in R^{3\times n}$ and $C_{{sc.m}_{\omega }}\in R^{3\times n}$ involve the Coriolis and centrifugal terms originating from the interaction between the arm and the spacecraft base.

The model presented in this article has been extensively used in simulations to test the controllers presented in Seddaoui et al. (2019) and Seddaoui and Saaj (2019). The controllers are model-based controllers designed as a combined *H*_*∞*_ controller (Seddaoui and Saaj, 2019) and its extension as an adaptive controller to minimize the control forces and torques (Seddaoui et al., 2019). Moreover, the CFSR model and the adaptive *H*_*∞*_ controller were used to test the trajectory planning algorithm introduced by Seddaoui and Saaj (2021). Simulation results showcasing the desired and actual spacecraft position and orientation trajectories, the desired and actual trajectories of the arm joints, and the final Cartesian motion of the CFSR when using the model presented in this article are presented by Seddaoui and Saaj (2021).

## 7 Conclusion

An accurate mathematical model that captures the complex dynamics of a space robot is helpful for effective pose control of space robot in extreme conditions. The controlled-floating mode of operation of a space robot is distinct from the free-flying and free-floating modes in terms of the nature of motion and corresponding system model. This tutorial guides the reader through a systematic process to derive the nonlinear dynamic model of CFSR. The equation of motion derived with respect to a frame attached to the target spacecraft complies with the close-proximity relative motion in orbit. The changes in the CoM and subsequent changes to the overall inertia matrix of the coupled system were considered. The refined model of the CFSR presented includes a few additional mathematical terms that account for these perturbations. In short, this tutorial helps users to compute the CFSR model as accurately and efficiently as possible.

It is important to note that the model presented here can be used for space robots of any size and mass. This is possible because the physical parameters of the space robot are the inputs to the generated matrices, which are the building blocks of the final model. One exception is the array of rotation matrices specific to each space robot as it needs to be defined beforehand. Unlike free-flying and free-floating space robots, a CFSR will require an advanced path-planning algorithm to generate optimal trajectories for both the arm and its base. This path planner must consume as little energy as possible and produce a minimal dynamic coupling effect. Moreover, a controller capable of executing the desired motion with a small control effort is also vital for the mission’s success.

The equation of motion of the controlled-floating space robot cannot be reduced to that of the freefloating space robot because they are fundamentally different. The latter is based on the generalized Jacobian matrix that is specific to free-floating robots with an assumed fixed CoM. In this method, the dynamics and kinematics of the space robot are tightly related and part of the same equation that is the generalized Jacobian matrix. Alternatively, the free-flying mode could be a subset of the CFSR when only controlled translation of the base spacecraft is required without the need to change its attitude. This state is different from free-floating mode where both translation and rotation of the base spacecraft are uncontrolled. This scenario is possible theoretically, but more research is needed to verify this statement, which is outside the scope of this article.

## Data Availability

The original contributions presented in the study are included in the article/Supplementary Material, further inquiries can be directed to the corresponding author.
