# Supplementary material for: Modeling a Controlled-Floating Space Robot for In-Space Services: A Beginner’s Tutorial
Source: Front Robot AI. 2021 Dec 24;8:725333. doi: 10.3389/frobt.2021.725333 (PMC8739970; doi:10.3389/frobt.2021.725333)
Supplement: Supplementary file 1 [file DataSheet1.pdf]

Modeling a Controlled-Floating Space Robot for In-Space Services: A Beginner's Tutorial

Asma Seddaoui [1], Chakravathini Mini Saaj [2,*] and Manu Harikrishnan Nair [2]

[1] *Surrey Space Centre, Department of Electronic and Electrical Engineering, University of Surrey, Guildford, United Kingdom*

[2] *Lincoln Centre for Autonomous Systems (L-CAS), University of Lincoln, Lincoln, United Kingdom*

Correspondence*:
Chakravathini Mini Saaj
MSaaj@lincoln.ac.uk

Appendix

This appendix presents detailed derivations of some vectors involved in the mathematical model for the dynamics and kinematics of the CFSR. Some of these vectors are illustrated in Fig. 5.

Angular momentum

The angular momentum of a multi-body system is defined as follows:

$$\boldsymbol{\mathcal{L}} = \sum_{i=0}^{n} \boldsymbol{r_i} \times m_i \dot{\boldsymbol{r}}_{\boldsymbol{i}}. \tag{71}$$

Taking $\boldsymbol{r_i} = \boldsymbol{r_\xi} + \boldsymbol{r_{i\xi}}$ and $\dot{\boldsymbol{r}}_{\boldsymbol{i}} = \dot{\boldsymbol{r}}_{\boldsymbol{\xi}} + \dot{\boldsymbol{r}}_{\boldsymbol{i\xi}} + \boldsymbol{\omega_{\xi B}} \times \boldsymbol{r_{i\xi}}$, Eq. (71) becomes:

$$\left.\begin{aligned} \boldsymbol{\mathcal{L}} &= \textstyle\sum_{i=0}^{n} \left(\left(\boldsymbol{r_\xi} + \boldsymbol{r_{i\xi}} \right) \times m_i \left(\dot{\boldsymbol{r}}_{\boldsymbol{\xi}} + \dot{\boldsymbol{r}}_{\boldsymbol{i\xi}} + \boldsymbol{\omega_{\xi B}} \times \boldsymbol{r_{i\xi}} \right) \right) \\ &= \textstyle\sum_{i=0}^{n} \left(m_i \boldsymbol{r_\xi} \times \dot{\boldsymbol{r}}_{\boldsymbol{\xi}} + m_i \boldsymbol{r_\xi} \times \dot{\boldsymbol{r}}_{\boldsymbol{i\xi}} - m_i \boldsymbol{r_{\xi B}} \times \boldsymbol{r_{i\xi}} \times \boldsymbol{\omega_{\xi B}} + \right. \\ &\quad \left. + m_i \boldsymbol{r_{i\xi}} \times \dot{\boldsymbol{r}}_{\boldsymbol{\xi}} + m_i \boldsymbol{r_{i\xi}} \times \dot{\boldsymbol{r}}_{\boldsymbol{i\xi}} - m_i \boldsymbol{r_{i\xi}} \times \boldsymbol{r_{i\xi}} \times \boldsymbol{\omega_{\xi B}} \right) \end{aligned}\right\}. \tag{72}$$

The terms $\sum_{i=0}^{n} m_i \boldsymbol{r_{\xi B}} \times \boldsymbol{r_{i\xi}} \times \boldsymbol{\omega_{\xi B}}$ and $\sum_{i=0}^{n} m_i \boldsymbol{r_{i\xi}} \times \dot{\boldsymbol{r}}_{\boldsymbol{\xi}}$ on the right hand side of Eq. (72) disappear as $\sum_{i=0}^{n} m_i \boldsymbol{r_{i\xi}} = \boldsymbol{0}$. This is proved as follows:

$$\left.\begin{aligned} \textstyle\sum_{i=0}^{n} m_i \boldsymbol{r_{i\xi}} &= \textstyle\sum_{i=0}^{n} m_i \left(\boldsymbol{r_i} - \boldsymbol{r_\xi} \right) \\ &= \textstyle\sum_{i=0}^{n} m_i \boldsymbol{r_i} - \sum_{i=0}^{n} m_i \boldsymbol{r_\xi} \\ &= \textstyle\sum_{i=0}^{n} m_i \boldsymbol{r_i} - M_t \boldsymbol{r_\xi} \\ &= \boldsymbol{0} \end{aligned}\right\}. \tag{73}$$

Similarly, the term $\sum_{i=0}^{n} m_i \boldsymbol{r_{\xi B}} \times \boldsymbol{\dot{r}_{i\xi}}$ on the right hand side of Eq. (72) disappears as $\sum_{i=0}^{n} m_i \boldsymbol{\dot{r}_{i\xi}} = \mathbf{0}$, which is proved as follows:

$$\left.\begin{array}{rcl} \sum_{i=0}^{n} m_i \boldsymbol{\dot{r}_{i\xi}} & = & \sum_{i=0}^{n} m_i \left(\boldsymbol{\dot{r}_i} - \boldsymbol{\dot{r}_\xi}\right) \\ & = & \sum_{i=0}^{n} m_i \boldsymbol{\dot{r}_i} - \sum_{i=0}^{n} m_i \boldsymbol{\dot{r}_\xi} \\ & = & \sum_{i=0}^{n} m_i \boldsymbol{\dot{r}_i} - M_t \boldsymbol{\dot{r}_\xi} \\ & = & \mathbf{0} \end{array}\right\}. \tag{74}$$

Taking into account Eqs. (73) and (74), the angular momentum describe in Eq. (71) becomes:

$$\left.\begin{array}{rcl} \mathcal{L} & = & \sum_{i=0}^{n} \left(m_i \boldsymbol{r_\xi} \times \boldsymbol{\dot{r}_\xi} + m_i \boldsymbol{r_{i\xi}} \times \boldsymbol{\dot{r}_{i\xi}} - m_i \boldsymbol{r_{i\xi}} \times \boldsymbol{r_{i\xi}} \times \boldsymbol{\omega_{\xi B}}\right) \\ & = & M_t \boldsymbol{r_\xi} \times \boldsymbol{\dot{r}_\xi} + \sum_{i=1}^{n} m_i \boldsymbol{r_{i\xi}} \times \boldsymbol{\dot{r}_{i\xi}} + \sum_{i=1}^{n} \mathbf{I_i} \boldsymbol{\omega_{\xi B}} \end{array}\right\}. \tag{75}$$

Expressing the angular momentum defined by Eq. (75) in terms of inertia tensor and angular velocity gives:

$$\left.\begin{array}{rcl} \mathcal{L} & = & \frac{r_\xi^2}{r_\xi^2} \boldsymbol{r_\xi} \times M_t \boldsymbol{\dot{r}_\xi} + \sum_1^n \frac{r_{i\xi}^2}{r_{i\xi}^2} \boldsymbol{r_{i\xi}} \times m_i \boldsymbol{\dot{r}_{i\xi}} + \sum_{i=1}^{n} \mathbf{I_i} \boldsymbol{\omega_{\xi B}} \\ & = & r_\xi^2 M_t \left(\frac{\boldsymbol{r_\xi} \times \boldsymbol{\dot{r}_\xi}}{r_\xi^2}\right) + \sum_1^n r_{i\xi}^2 m_i \left(\frac{\boldsymbol{r_{i\xi}} \times \boldsymbol{\dot{r}_{i\xi}}}{r_{i\xi}^2}\right) + \sum_{i=1}^{n} \mathbf{I_i} \boldsymbol{\omega_{\xi B}} \\ \mathcal{L} & = & \mathbf{I}_\xi \boldsymbol{\omega_\xi} + \sum_{i=1}^{n} \mathbf{I_i} \boldsymbol{\omega_i} + \sum_{i=1}^{n} \mathbf{I_i} \boldsymbol{\omega_{\xi B}} \end{array}\right\}. \tag{76}$$

Expressing the last term on the right hand side of Eq. (76) in terms of inertia tensors with respect to the $\sum_B$, using the parallel axis theorem, gives:

$$\mathcal{L} = \mathbf{I}_\xi \boldsymbol{\omega_\xi} + \sum_{i=1}^{n} \mathbf{I_i} \boldsymbol{\omega_i} + \sum_{i=1}^{n} \left(\mathbf{I_i} - m_i [\boldsymbol{r_{iB}}]_\times [\boldsymbol{r_{iB}}]_\times \boldsymbol{\omega_{\xi B}}\right). \tag{77}$$

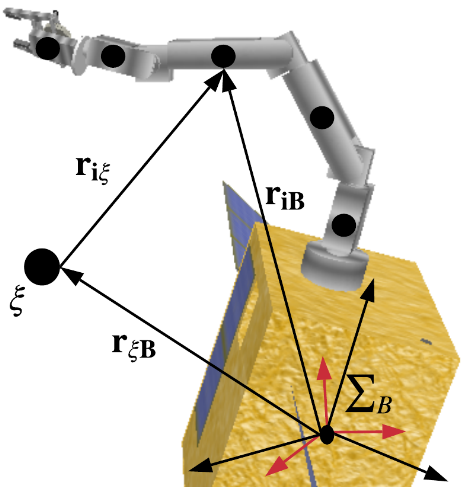

Figure 5. Artistic illustration depicting the vectors involved in the derivation of the angular momentum (Seddaoui, 2020)

The CoM position vector and its derivative

The position vector from the origin of $\sum_B$ to the CoM ξ, in $\sum_T$, is defined as follows:

$$\left.\begin{array}{rcl} \boldsymbol{r_{\xi B}} & = & \dfrac{1}{M_t}\sum_{i=1}^{n} m_i \boldsymbol{r_{iB}} \\ & = & \dfrac{1}{M_t}\sum_{i=1}^{n} m_i \left(\mathbf{R_{L_i}}\boldsymbol{b_i} + \mathbf{R_{L_{i-1}}}\boldsymbol{s_{i-1}}\right) \end{array}\right\}. \tag{78}$$

The derivative of $\boldsymbol{r_{\xi B}}$, described by Eq. (78), is:

$$\dot{\boldsymbol{r}}_{\boldsymbol{\xi B}} = \frac{1}{M_t}\sum_{i=1}^{n} m_i \left(\dot{\mathbf{R}}_{\mathbf{L_i}}\boldsymbol{b_i} + \dot{\mathbf{R}}_{\mathbf{L_{i-1}}}\boldsymbol{s_{i-1}}\right). \tag{79}$$

The derivative of the overall inertia tensor

The derivative of the inertia tensor $\mathbf{I}_\xi$ is defined as follows:

$$\left.\begin{array}{rcl} \dot{\mathbf{I}}_\xi & = & M_t[\dot{\boldsymbol{r}}_{\boldsymbol{\xi B}}]_\times[\boldsymbol{r_{\xi B}}]_\times + M_t[\boldsymbol{r_{\xi B}}]_\times[\dot{\boldsymbol{r}}_{\boldsymbol{\xi B}}]_\times + \dot{\mathbf{I}}_{\mathbf{iB}} \\ \dot{\mathbf{I}}_{\mathbf{iB}} & = & \sum_{i=1}^{n} m_i[\dot{\boldsymbol{r}}_{\boldsymbol{iB}}]_\times[\boldsymbol{r_{iB}}]_\times + \sum_{i=1}^{n} m_i[\boldsymbol{r_{iB}}]_\times[\dot{\boldsymbol{r}}_{\boldsymbol{iB}}]_\times \end{array}\right\}. \tag{80}$$

References

Seddaoui, A. (2020). *Precise Motion Control of a Space Robot for In-orbit Close Proximity Manoeuvres*. Ph.D. thesis, University of Surrey.
