# Supplementary material for: Modeling a Controlled-Floating Space Robot for In-Space Services: A Beginner’s Tutorial
Source: Front Robot AI. 2021 Dec 24;8:725333. doi: 10.3389/frobt.2021.725333 (PMC8739970; doi:10.3389/frobt.2021.725333)
Supplement: Supplementary file 2 [file Supplementary_Material.docx]

Appendix

This appendix presents detailed derivations of some vectors involved in the mathematical model for the dynamics and kinematics of the CFSR. Some of these vectors are illustrated in Fig. 5.

Angular momentum

The angular momentum of a multi-body system is defined as follows:

<display>\begin{equation} \boldsymbolmathcal{L} = \sum_{i=0}^{n} \boldsymbol{r_{i}} \times m_{i} \boldsymbol{\dot{r}_{i}}. \end{equation}</display>
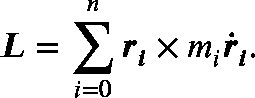
 (71)

Taking ***r_i_*** = ***r_ξ_*** + ***r_iξ_*** and <inline>\boldsymbol{\dot{r}_{i}} = \boldsymbol{\dot{r}_{\xi}} + \boldsymbol{\dot{r}_{i \xi}} + \boldsymbol{\omega_{\xi B}} \times \boldsymbol{r_{i \xi}} </inline>
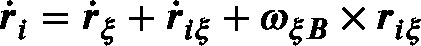
, Eq. (71) becomes:

<display>\begin{equation} \left. \begin{matrix} \boldsymbolmathcal{L} &= \sum_{i=0}^{n} \left( \left(\boldsymbol{r_{\xi}} + \boldsymbol{r_{i \xi}} \right) \times m_{i} \left(\boldsymbol{\dot{r}_{\xi}} + \boldsymbol{\dot{r}_{i \xi}} + \boldsymbol{\omega_{\xi B}} \times \boldsymbol{r_{i \xi}} \right) \right) \\ &= \sum_{i=0}^{n} \left(m_{i} \boldsymbol{r_{\xi}} \times \boldsymbol{\dot{r}_{\xi}} + m_{i} \boldsymbol{r_{\xi}} \times \boldsymbol{\dot{r}_{i \xi}} - m_{i} \boldsymbol{r_{\xi B}} \times \boldsymbol{r_{i \xi}} \times \boldsymbol{\omega_{\xi B}} \; + \right.\\ & \left. + \; m_{i} \boldsymbol{r_{i \xi}} \times \boldsymbol{\dot{r}_{\xi}} + m_{i} \boldsymbol{r_{i \xi}} \times \boldsymbol{\dot{r}_{i \xi}} - m_{i} \boldsymbol{r_{i \xi}} \times \boldsymbol{r_{i \xi}} \times \boldsymbol{\omega_{\xi B}} \right) \end{matrix} \right\}. \end{equation}</display>
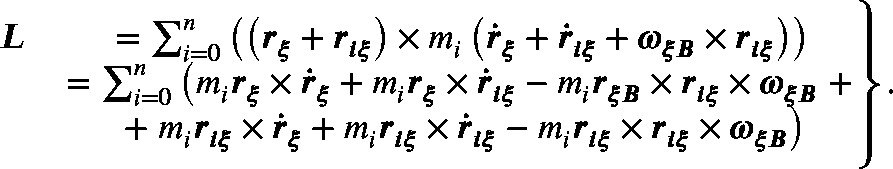
 (72)

The terms <inline>\sum_{i=0}^{n} m_{i} \boldsymbol{r_{\xi B}} \times \boldsymbol{r_{i \xi}} \times \boldsymbol{\omega_{\xi B}}</inline>
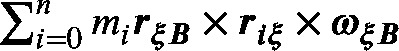
 and <inline> \sum_{i=0}^{n} m_{i} \boldsymbol{r_{i \xi}} \times \boldsymbol{\dot{r}_{\xi}}</inline>
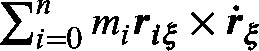
 on the right hand side of Eq. (72) disappear as <inline>\sum_{i=0}^{n} m_{i} \boldsymbol{r_{i \xi}} = \mathbf{0}</inline>
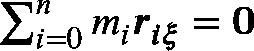
. This is proved as follows:

<display>\begin{equation} \left. \begin{matrix} \sum_{i=0}^{n} m_{i} \boldsymbol{r_{i \xi}} &= \sum_{i=0}^{n} m_{i} \left(\boldsymbol{r_{i}} - \boldsymbol{r_{\xi}} \right) \\ &= \sum_{i=0}^{n} m_{i} \boldsymbol{r_{i}} - \sum_{i=0}^{n} m_{i} \boldsymbol{r_{\xi}} \\ &= \sum_{i=0}^{n} m_{i} \boldsymbol{r_{i}} - M_{t} \boldsymbol{r_{\xi}} \\ &= \mathbf{0} \end{matrix} \right\}. \end{equation}</display>
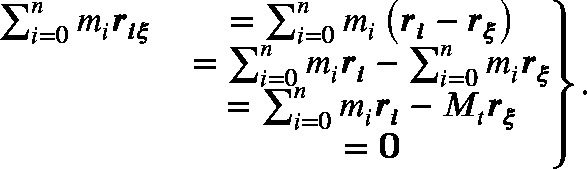
 (73)

Similarly, the term <inline> \sum_{i=0}^{n} m_{i} \boldsymbol{r_{\xi B}} \times \boldsymbol{\dot{r}_{i \xi}}</inline>
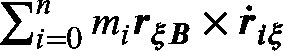
 on the right hand side of Eq. (72) disappears as <inline>\sum_{i=0}^{n} m_{i} \boldsymbol{\dot{r}_{i \xi}} = \mathbf{0}</inline>
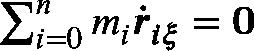
, which is proved as follows:

<display>\begin{equation} \left. \begin{matrix} \sum_{i=0}^{n} m_{i} \boldsymbol{\dot{r}_{i \xi}} &= \sum_{i=0}^{n} m_{i} \left(\boldsymbol{\dot{r}_{i}} - \boldsymbol{\dot{r}_{\xi}} \right) \\ &= \sum_{i=0}^{n} m_{i} \boldsymbol{\dot{r}_{i}} - \sum_{i=0}^{n} m_{i} \boldsymbol{\dot{r}_{\xi}} \\ &= \sum_{i=0}^{n} m_{i} \boldsymbol{\dot{r}_{i}} - M_{t} \boldsymbol{\dot{r}_{\xi}} \\ &= \mathbf{0} \end{matrix} \right\}. \end{equation}</display>
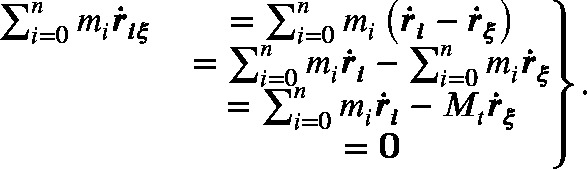
 (74)

Taking into account Eqs. (73) and (74), the angular momentum describe in Eq. (71) becomes:

<display>\begin{equation} \left. \begin{matrix} \boldsymbolmathcal{L} &= \sum_{i=0}^{n} \left( m_{i} \boldsymbol{r_{\xi}} \times \boldsymbol{\dot{r}_{\xi}} + m_{i} \boldsymbol{r_{i \xi}} \times \boldsymbol{\dot{r}_{i \xi}} - m_{i} \boldsymbol{r_{i \xi}} \times \boldsymbol{r_{i \xi}} \times \boldsymbol{\omega_{\xi B}} \right) \\ &= M_{t} \boldsymbol{r_{\xi}} \times \boldsymbol{\dot{r}_{\xi}} + \sum_{i=1}^{n} m_{i} \boldsymbol{r_{i \xi}} \times \boldsymbol{\dot{r}_{i \xi}} + \sum_{i=1}^{n} \mathbf{I}_{\mathbf{i}} \boldsymbol{\omega_{\xi B}} \end{matrix} \right\}. \end{equation}</display>
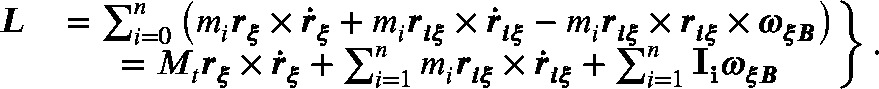
 (75)

Figure 5. Artistic illustration depicting the vectors involved in the derivation of the angular momentum (Seddaoui, 2020)

Expressing the angular momentum defined by Eq. (75) in terms of inertia tensor and angular velocity gives:

<display>\begin{equation}\\ \left. \begin{matrix} \boldsymbolmathcal{L} &= \dfrac{r_{\xi}^{2}}{r_{\xi}^{2}} \boldsymbol{r_{\xi}} \times M_{t} \boldsymbol{\dot{r}_{\xi}} + \sum_{1}^{n} \dfrac{r_{i \xi}^{2}}{r_{i \xi}^{2}} \boldsymbol{r_{i \xi}} \times m_{i} \boldsymbol{\dot{r}_{i \xi}} + \sum_{i=1}^{n} \mathbf{I}_{\mathbf{i}} \boldsymbol{\omega_{\xi B}} \\ &= r_{\xi}^{2} M_{t} \left(\dfrac{\boldsymbol{r_{\xi}} \times \boldsymbol{\dot{r}_{\xi}}}{r_{\xi}^{2}} \right) + \sum_{1}^{n} r_{i \xi}^{2} m_{i} \left(\dfrac{\boldsymbol{r_{i \xi}} \times \boldsymbol{\dot{r}_{i \xi}}}{r_{i \xi}^{2}} \right) + \sum_{i=1}^{n} \mathbf{I}_{\mathbf{i}} \boldsymbol{\omega_{\xi B}} \\ \boldsymbolmathcal{L} &= \mathbf{I}_{\boldsymbol{\upxi}} \boldsymbol{\omega_{\xi}} + \sum_{i=1}^{n} \mathbf{I}_{\mathbf{i}} \boldsymbol{\omega_{i}} + \sum_{i=1}^{n} \mathbf{I}_{\mathbf{i}} \boldsymbol{\omega_{\xi B}} \end{matrix} \right\}. \end{equation}</display> (76)

Expressing the last term on the right hand side of Eq. (76) in terms of inertia tensors with respect to the *∑_B_*, using the parallel axis theorem, gives:

<display>\begin{equation}\\ \boldsymbolmathcal{L} = \mathbf{I}_{\boldsymbol{\upxi}} \boldsymbol{\omega_{\xi}} + \sum_{i=1}^{n} \mathbf{I}_{\mathbf{i}} \boldsymbol{\omega_{i}} + \sum_{i=1}^{n} \left(\mathbf{I}_{\mathbf{i}} - m_{i} [\boldsymbol{r_{iB}}]_{\times} [\boldsymbol{r_{iB}}]_{\times} \boldsymbol{\omega_{\xi B}} \right). \end{equation}</display>
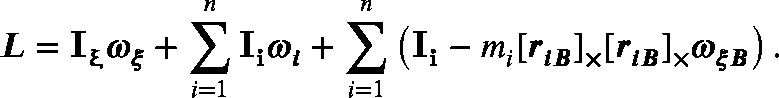
 (77)

The CoM position vector and its derivative

The position vector from the origin of *∑_B_* to the CoM *ξ*, in *∑_T_*, is defined as follows:

<display>\begin{equation}\\ \left. \begin{matrix} \boldsymbol{r_{\xi B}} &= \dfrac{1}{M_{t}} \sum_{i=1}^{n} m_{i}\boldsymbol{r_{iB}} \\ &= \dfrac{1}{M_{t}} \sum_{i=1}^{n} m_{i} \left( \mathbf{R}_{\mathbf{L}_{\mathbf{i}}} \boldsymbol{b_{i}} + \mathbf{R}_{\mathbf{L}_{\mathbf{i}-\mathbf{1}}} \boldsymbol{s_{i-1}} \right) \end{matrix} \right\}. \end{equation}</display>
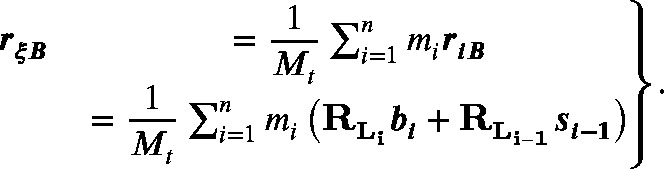
 (78)

The derivative of ***r_ξB_***, described by Eq. (78), is:

<display>\begin{equation} \boldsymbol{\dot{r}_{\xi B}} = \dfrac{1}{M_{t}} \sum_{i=1}^{n} m_{i} \left( \dot{\mathbf{R}}_{\mathbf{L}_{\mathbf{i}}} \boldsymbol{b_{i}} + \dot{\mathbf{R}_{L_{i-1}}} \boldsymbol{s_{i-1}} \right). \end{equation}</display>
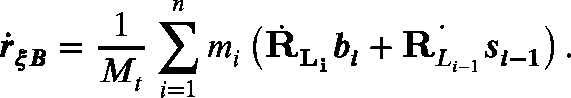
 (79)

The derivative of the overall inertia tensor

The derivative of the inertia tensor **I*_ξ_*** is defined as follows:

<display>\begin{equation} \left. \begin{matrix} \dot{\mathbf{I}}_{\boldsymbol{\upxi}} &= M_{t} [\boldsymbol{\dot{r}_{\xi B}}]_{\times} [\boldsymbol{r_{\xi B}}]_{\times} + M_{t} [\boldsymbol{r_{\xi B}}]_{\times} [\boldsymbol{\dot{r}_{\xi B}}]_{\times} + \dot{\mathbf{I}}_{\mathbf{iB}} \\ \dot{\mathbf{I}}_{\mathbf{iB}} &= \sum_{i=1}^{n} m_{i} [\boldsymbol{\dot{r}_{iB}}]_{\times} [\boldsymbol{r_{iB}}]_{\times} + \sum_{i=1}^{n} m_{i} [\boldsymbol{r_{iB}}]_{\times} [\boldsymbol{\dot{r}_{iB}}]_{\times} \end{matrix} \right\}. \end{equation}</display>
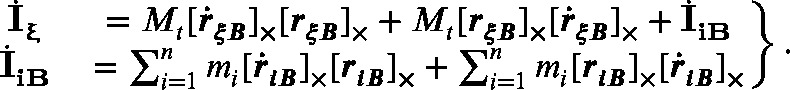
 (80)
